# FTO-mediated LINC01134 stabilization to promote chemoresistance through miR-140-3p/WNT5A/WNT pathway in PDAC

**DOI:** 10.1038/s41419-023-06244-7

**Published:** 2023-11-01

**Authors:** Jin Lu, Yongsheng Yang, Xiangliang Liu, Xiao Chen, Wei Song, Zefeng Liu

**Affiliations:** 1 Cancer Center, The First Hospital of Jilin University, 71 Xinmin Street, Changchun, 130021 China; 2 Department of Hepatobiliary Pancreatic Surgery, The Second Hospital of Jilin University, 218 Ziqiang Street, Changchun, 130041 China; 3 Jilin Engineering Laboratory for Translational Medicine of Hepatobiliary and Pancreatic Diseases, Changchun, 130041 China

**Keywords:** Tumour biomarkers, Pancreatic cancer

## Abstract

Pancreatic ductal adenocarcinoma (PDAC) is a highly aggressive cancer most frequently detected at an advanced stage that limits treatment options to systemic chemotherapy, which has provided only marginal positive clinical outcomes. Currently, the first-line chemotherapeutic agent for PDAC is gemcitabine (GEM). However, the chemotherapy resistance to GEM is often overlooked in the clinical treatment of PDAC due to the lack of effective biological markers. Therefore, it is crucial to find new prognostic markers and therapeutic targets for patients with PDAC. In this study, we identified a novel regulatory mechanism in the development of resistance to GEM in PDAC. Here, we report that LINC01134 was significantly upregulated in primary tumors from PDAC patients. In vitro and in vivo functional studies revealed that LINC01134 promotes PDAC resistance to GEM through facilitating stem cell features and modulating the cell cycle. Mechanistically, LINC01134 interactes with tumor suppressor miR-497-5p in PDAC cells. Increased LINC01134 downregulates miR-140-3p to promotes the oncogenic WNT5A expression. Moreover, m^6^A demethylase FTO participated in the upregulation of LINC01134 by maintaining LINC01134 mRNA stability through YTHDF2. Taken together, the present study suggested FTO-mediated LINC01134 stabilization to promote chemotherapy resistance to GEM through miR-140-3p/WNT5A/WNT pathway in PDAC. Our study identified new prognostic markers and new therapeutic targets for patients with PDAC.

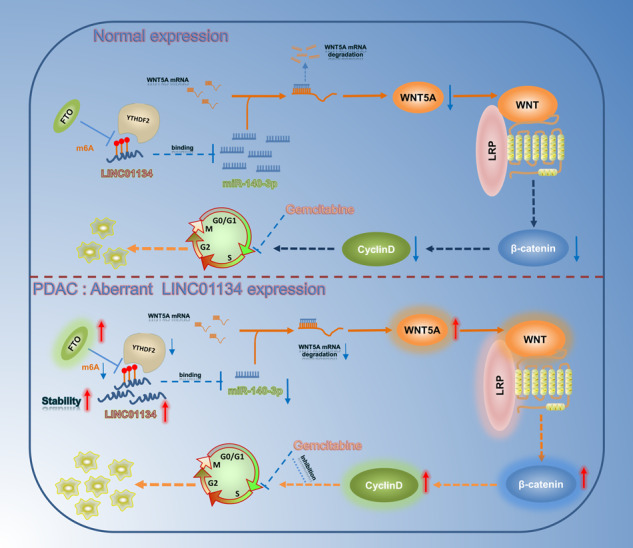

## Introduction

The pancreatic ductal adenocarcinoma (PDAC) is most common pathological type of pancreatic cancer accounting for approximately 90% of cases [1]. Pancreatic cancer, one of the most malignant solid tumors with an extremely poor prognosis, is the seventh leading cause of cancer-related death worldwide [2] and the sixth leading cause of cancer-related death in China [3, 4]. Although the current best treatment for PDAC is radical surgery, most cases have lost the opportunity for surgery when the tumor is detected, due to the lack of typical clinical manifestations in the early stages of PDAC [5]. Even if the patients can receive surgery timely, the postoperative recurrence and metastasis rates of patients with PDAC after surgery are very high [6].

According to a recent study that statistically investigated the prognosis of PDAC, the prognosis of PDAC is extremely poor with an overall 5-year survival rate of less than 10% and a median survival of approximately 6 months [6, 7]. Therefore, chemotherapy remains an irreplaceable part of the comprehensive treatment of PDAC. The current first-line chemotherapeutic agent for PDAC is gemcitabine (GEM), approved alone or in combination with other chemotherapeutic agents for the treatment of advanced PDAC [8]. However, the insensitivity of PDAC to most chemotherapeutic agents renders chemotherapy for PDAC ineffective. Therefore, it is of great clinical significance to investigate the occurrence and development mechanisms of PDAC in-depth and various genetic and epigenetic mechanisms involved in the development of GEM resistance to develop new effective targets and therapeutic strategies for the treatment of PDAC.

Long non-coding RNAs (lncRNAs) represent a group of RNAs with over 200 nucleotides that do not encode proteins or encode only short polypeptides [9]. An increasing number of studies have shown that lncRNAs play important roles in various biological processes, such as cell growth, differentiation, apoptosis, and migration [10–12]. lncRNAs exert their biological functions mainly by interacting with chromatin DNA, proteins, and other RNAs [13]. In addition, a large number of studies have confirmed that lncRNAs play an important role in tumorigenesis and development by regulating tumor cell proliferation, metabolism, invasion, migration, and stem cell differentiation [14–17]. Several lncRNAs have been considered as potential biological markers and therapeutic targets for the diagnosis and treatment of tumors [18]. Long intergenic non-protein-coding RNA 1134 (LINC01134) is a newly discovered lncRNA that has been studied for its role in the pathogenesis of hepatocellular carcinoma [19–22]. However, the role of LINC01134 in other tumors remains largely unknown, especially in PDAC. To the best of our knowledge, no studies have been published investigating the role of LINC01134 in PDAC.

In this study, we identified LINC01134 as a prognostic marker for predicting the efficacy of GEM for the treatment of PDAC based on the pharmacological effects of GEM cytotoxic activity. We found that LINC01134 was highly expressed in the GEM-resistant patient-derived xenografts (PDX) model and was positively correlated with the G1/S-specific cyclin as well as cell cycle protein-dependent kinase with a statistical significance. In in vivo and in vitro experiments, we observed that LINC01134 promotes the stem cell characteristics of PDAC cells and the proliferation of PDAC cells by facilitating their conversion from G0/G1 to S-phase, a mechanism that antagonizes the cytotoxic activity of GEM and leads to the development of GEM resistance in PDAC cells. Further studies indicated that LINC01134 can regulate the WNT pathway by competitively binding to miR-140-3p, affecting its targeting to WNT5A. In addition, the application of WNT pathway inhibitors reverses the drug resistance of PDAC cells to GEM. Our study suggests that LINC01134/miR -140-3p/WNT5A/WNT pathway can serve as a new key axis for regulating the resistance of GEM in PDAC.

## Results

### LINC01134 is significantly upregulated in PDAC and indicates poor prognosis

**Fig. 1:**
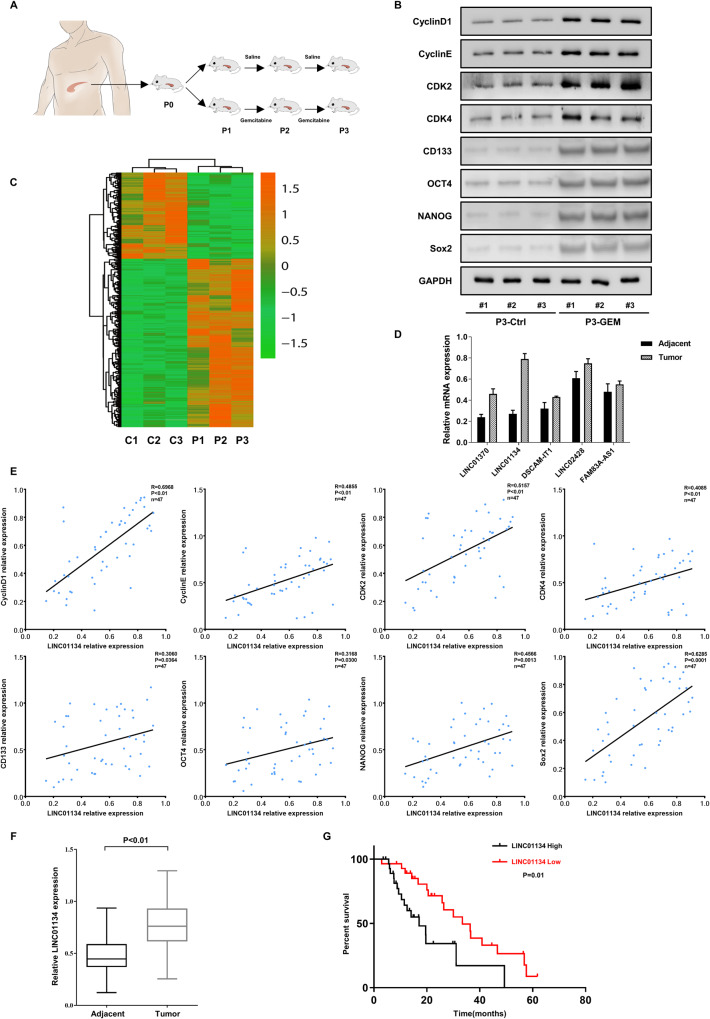
LINC01134 is upregulated in PDAC. **A** Experimental procedure of the GEM-treated PDX PDAC model. **B** Immunoblotting assay to detect the expression of cyclin and cyclin-dependent kinases (Cyclin D1, Cyclin E, CDK2, CDK4) at the G1/S cell cycle checkpoint and cancer stem cell master genes (CD133, OCT4, NANOG, and Sox2) in tissues from the 3-generation PDX mice that treated with saline or gemcitabine. **C** Heatmap shows differentially expressed lncRNAs in GEM-treated and control PDX mice. **D** Comparison of expression of lncRNAs in tumor tissues from 36 PDAC patients with paired paracancerous tissues. **E** Correlation analysis of LINC01134 with cyclin and cyclin-dependent kinases and cancer stem cell master genes as described in (**B**). **F** Comparison of the expression of LINC01134 in tumor tissues and paired paraneoplastic tissues from 70 PDAC patients. **G** Kaplan–Meier analysis of the correlation between expression levels of LINC01134 and PDAC prognosis in 57 patients. Data are expressed as mean ± SD *p < 0.05, **p < 0.01.

There is no doubt about the efficacy of GEM for the treatment of PDAC, and the sensitivity of PDAC cells to GEM is higher than that to other chemotherapeutic agents [23]. However, many patients begin to develop resistance to GEM within a few weeks after starting treatment, resulting in poor therapeutic outcomes [24]. To understand the mechanism of PDAC resistance to GEM, we first established a PDX model of GEM resistance. Surgically resected primary pancreatic cancer tissues were finely trimmed and transplanted directly into immunodeficient mice, and the tumor-bearing mice were treated with saline or GEM for several generations (Fig. 1A). Considering that the anti-tumor cell properties of GEM are mainly to block cell progression through the G1/S phase junction [25, 26], we examined the expression of cyclins and cyclin-dependent kinases at the G1/S cell cycle checkpoint in the P3-PDX of the GEM-treated and control groups. Figure 1B shows that the expression of cyclin and cyclin-dependent kinases (Cyclin D1, Cyclin E, CDK2, CDK4) and cancer stem cell master genes (CD133, OCT4, NANOG, and Sox2) in the GEM-treated group was significantly higher than that in the control group, which is consistent with our expectation. lncRNAs plays an important role in the modulation of cell cycle [10, 13], We then applied lncRNA sequencing to identify the differentially expressed lncRNAs between the GEM-treated and control groups of the P3-PDX (Fig. 1C) and selected the five most significantly upregulated lncRNAs for further study. qRT-PCR revealed that the expression of LINC01134 was significantly (*p* < 0.01) higher in 36 pairs of PDAC tissues than that in normal tissues adjacent to cancer (Fig. 1D). Correlation analysis showed that the abundance of LINC01134 in PDAC tissues was positively correlated with the levels of cyclin and cyclin-dependent kinases (Cyclin D1, Cyclin E, CDK2, CDK4) and cancer stem cell master genes (CD133, OCT4, NANOG, and Sox2) (Fig. 1E), and this result was more representative compared with the other four lncRNAs (Fig. S1A). qRT-PCR confirmed that the expression of LINC01134 was higher in P3-PDX in the GEM-treated group than that in the control group (Fig. S1B). Subsequently, we examined the expression of LINC01134 in 70 patients with PDAC and found that the expression of LINC01134 was significantly higher in PDAC tissues compared with normal tissues adjacent to cancer (Fig. 1F). Further correlation analysis with clinicopathological parameters showed that the high expression level of LINC01134 was correlated with the tumor size, local lymph node metastasis, and distant metastasis (Table S1). Kaplan–Meier analysis also showed that the overall survival was significantly lower in patients with high LINC01134 expression (Fig. 1G). The expression levels of LINC01134 were also investigated in a normal pancreatic cell line (HPDE6-C7) and seven pancreatic cancer cell lines (PANC-1, MIA PaCa-2, BxPC-3, SW1990, CFPAC-1, CaPan-1, APC-1). We found that the expression of LINC01134 in pancreatic cancer cell lines was significantly higher than that in the normal pancreatic cell line (Fig. S1C).These results suggested that LINC01134 was highly expressed in PDAC tissues and positively correlated with the malignancy of PDAC. In addition, the overexpression of LINC01134 was closely associated with cyclins and cyclin-dependent kinases at the G1/S cell cycle checkpoint, which may play an important role in the resistance to GEM in PDAC cells.

### Silencing LINC01134 suppresses stem cell features in PDAC cells

**Fig. 2:**
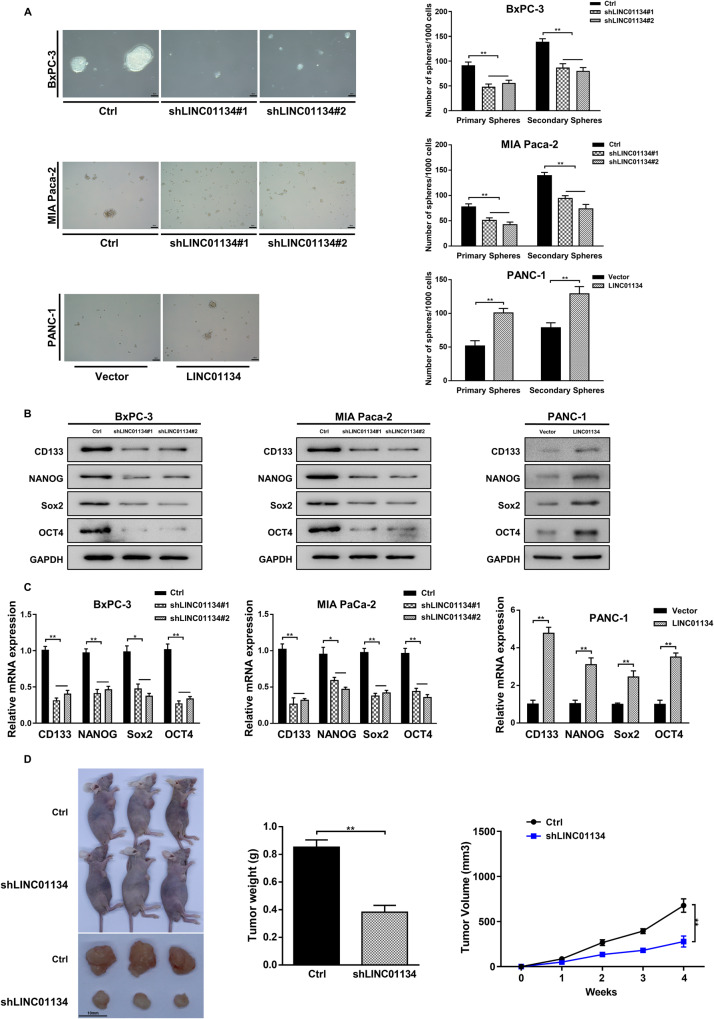
Silencing LINC01134 suppresses PDAC cells with stem cell properties, thereby inhibiting the PDAC development. **A** Statistical analysis of the primary and secondary sphere formation ability of PDAC cells, and representative images show secondary sphere formation in these cells. **B** Western blot was performed to detect the effects of silencing or overexpression of LINC01134 on the expression of the stemness-related genes. **C** qRT-PCR detects the effect of silencing or overexpression of LINC01134 on the expression of the stemness-related genes. **D** Effects of silencing LINC01134 on transplanted tumors in nude mice. Data are expressed as mean, *p < 0.05, **p < 0.01.

There is growing evidence that the stem cell features of malignancies are associated with their chemoresistance [27, 28]. Therefore, we investigated the effect of LINC01134 on the stem cell features of pancreatic cancer cells. We found that silencing LINC01134 resulted in decreased sphere formation in BxPC-3 and MIA PaCa-2 cells, while the overexpression of LINC01134 enhanced sphere formation in PANC-1 cells (Fig. 2A). We then investigated the potential regulatory effect of LINC01134 on the expression of CSC markers (CD133, NANOG, Sox2, OCT4). Results showed that silencing LINC01134 significantly inhibited the protein (Fig. 2B) and mRNA (Fig. 2C) expression of CD133, NANOG, Sox2, and OCT4, while the overexpression of LINC01134 promoted the expression of these CSC markers .

The results of in vivo experiments indicated that silencing LINC01134 can significantly reduce the tumorigenicity of PDAC cells (Fig. 2D). It showed that in the xenograft model, PDAC cells with LINC01134 silenced exhibited a slow and unsustainable tumorigenic process compared to the control group. Overall, these findings suggest that silencing of LINC01134 can arrest the G1/S cell cycle and significantly inhibits the stem cell features of PDAC cells, which in turn inhibits the tumorigenicity of PDAC cells both in vitro and in vivo.

### Silencing LINC01134 overcomes chemoresistance in PDAC cells by inhibiting stem cell features and arresting the cell cycle of PDAC

**Fig. 3:**
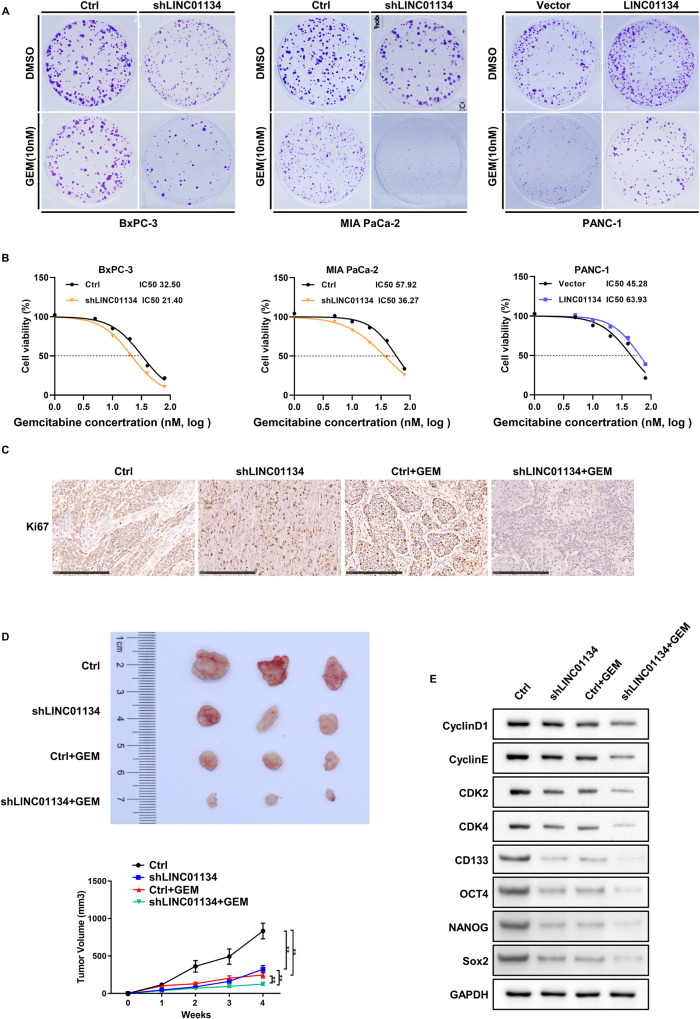
LINC01134 promotes the chemotherapy resistance of PDAC to GEM in vitro and in vivo. **A** Colony formation assay of cells containing silenced or overexpressed LINC01134 after GEM treatment. **B** Effects of different concentrations of GEM on the viability of PDAC cells. **C** Expression of Ki67 in xenograft tumors formed from PDAC cells transfected with Ctrl or shLINC01134 in GEM-treated nude mice. **D** Typical images of xenograft tumors formed from PDAC cells transfected with Ctrl or shLINC01134 in GEM-treated nude mice. Tumor growth curves of PDAC cells transfected with Ctrl or shLINC01134 in GEM-treated nude mice. **E** Expression of cyclin and cyclin-dependent kinases and and cancer stem cell master genes in xenograft tumor tissues formed from PDAC cells that transfected with Ctrl or shLINC01134 in GEM-treated nude mice. Data are expressed as mean, *p < 0.05, **p < 0.01.

Next, we investigated whether LINC01134 affects the chemosensitivity of PDAC cells to GEM. The colony formation assay showed that silencing LINC01134 can inhibit the proliferation of PDAC cells in vitro and enhance the chemosensitivity of PDAC cells to GEM, as evidenced by the decreased colony formation and reduced cell viability. In contrast, the overexpression of LINC01134 had the opposite effect on PDAC cells as shown in Fig. 3A and Fig. S3A, B. Results showed that silencing LINC01134 decreased the IC50 values of BxPC-3 and MIA PaCa-2 cells and enhanced the chemosensitivity of these two cell lines to GEM, whereas the overexpression of LINC01134 increased the IC50 values of PANC-1 cells and enhanced their resistance to GEM (Fig. 3B). The results from in vivo experiments are consistent with those from in vitro experiments. In the in vivo experiments, we found that silencing LINC01134 reduced the size and expression of Ki67 of transplanted tumors in saline (NS) and GEM-treated nude mice and enhanced the cytotoxic activity of GEM (Fig. 3C, D, Fig. S3D). Later, we examined the expression of key proteins at the G1/S cell cycle checkpoint in transplanted tumors using western blot. We found that both silencing of LINC01134 and the use of GEM resulted in reduced expression of Cyclin D1, Cyclin E, CDK2, CDK4, CD133, OCT4, NANOG and Sox2 at the protein level, and the combination of both further reduced the expression of those proteins (Fig. 3E).

To investigate the role of LINC01134 on PDAC resistance to GEM, we transfected shLINC01134 and LINC01134 mimics lentiviruses into pancreatic cancer cell lines with high levels of endogenous LINC01134 (BxPC-3, MIA PaCa-2) and the cell line with relatively low expression LINC01134 (PANC-1), respectively. The transfection efficiency was validated using qRT-PCR (Fig. S2A). To investigate the effect of LINC01134 on the pancreatic cancer cell cycle, we used flow cytometry to perform cell cycle analysis, which suggested that silencing LINC01134 triggered G1/S cell cycle arrest in BxPC-3 cells and MIA PaCa-2 cells (Fig. S2B), whereas the overexpression of LINC01134 had the opposite effect on PANC-1 cells (Fig. S2B). We then investigated the effect of LINC01134 on the expression of key proteins at the G1/S cell cycle checkpoint by western blot and showed that silencing of LINC01134 resulted in a reduced amount of Cyclin D1, Cyclin E, CDK2, and CDK4 in BxPC-3 cells and MIA PaCa-2 cells at the protein level, while the overexpression of LINC01134 resulted in increased levels of these proteins in PANC-1 cells (Fig. S2C).

In summary, silencing of LINC01134 overcomes chemoresistance in PDAC cells by inhibiting stem cell features and arresting the cell cycle of PDAC

### LINC01134 upregulates WNT5A expression through competitively binding to miR-140-3p

**Fig. 4:**
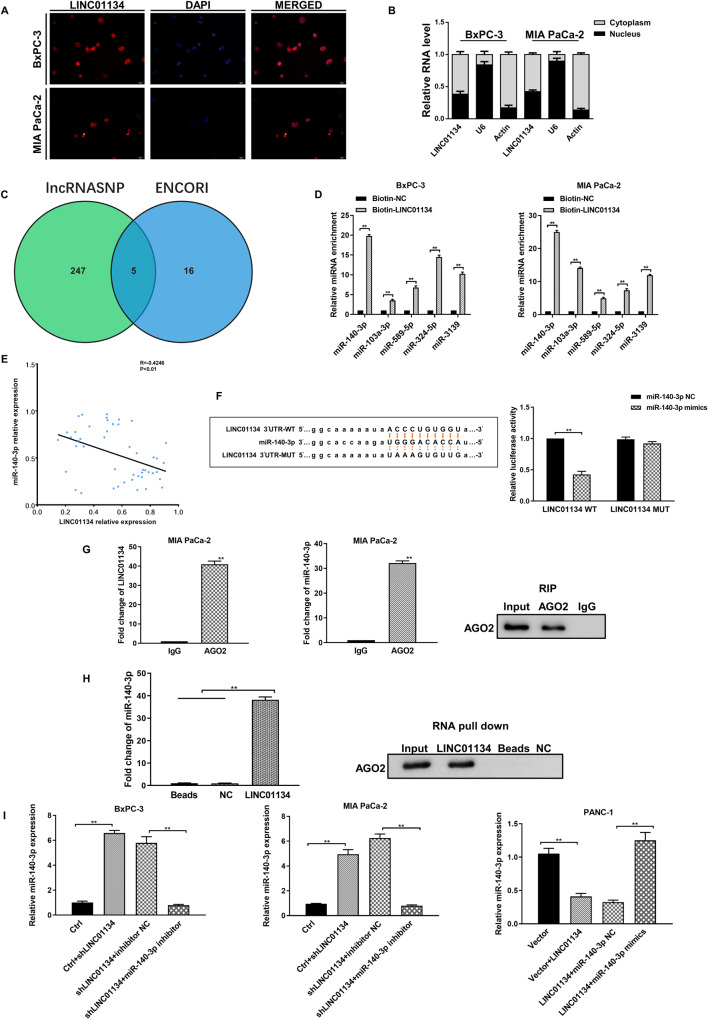
LINC01134 acts as a molecular sponge for miR-140-3p. **A** FISH assay detects the localization of LINC01134 in PDAC cells. **B** qRT-PCR detects the distribution of LINC01134 in nuclear and cytoplasm in PDAC cells. **C** Wayne diagram shows microRNAs predicted by the bioinformatics databases Encyclopedia of RNA Interactomes and lncRNASNP2 to target bind to LINC01134. **D** RNA pull-down analysis shows that miR-140-3p was highly enriched in LINC01134 precipitates. **E** Correlation analysis shows a significant negative correlation between LINC01134 and miR-140-3p in PDAC. **F** Dual-luciferase reporter assay detects the relative activity of luciferase in transfected PDAC cells. **G** RIP assay detects the relative enrichment of LINC01134 and miR-140-3p in anti-IgG or anti-AGO2 specific immunoprecipitates. **H** RNA pull-down assay was used to detect the inter-relationship between LINC01134, miR-140-3p, and AGO2. **I** Effects of silencing or overexpression of LINC01134 on the expression of miR-140-3p. Data are expressed as mean, *p < 0.05, **p < 0.01.

The mechanisms by which lncRNAs regulate the biological functions of malignant tumors often depend on their subcellular localization [29, 30]. We investigated the subcellular distribution of LINC01134 in PDAC cells by FISH and qRT-PCR. As shown in Fig. 4A, B, there was a higher level of LINC01134 expressed in the cytoplasm. In addition, lncRNAs in the cytoplasm are usually believed to bind microRNAs competitively through the ceRNA mechanism, thereby reducing the regulatory effect of microRNAs on target genes [31]. We hypothesized that LINC01134 also acts through the ceRNA mechanism in pancreatic cancer.

To test this hypothesis, we used the bioinformatics databases Encyclopedia of RNA Interactomes (ENCORE, previously known as starBase v2.0) and lncRNASNP2 (http://bioinfo.life.hust.edu. CN) to predict the target binding capabilities of microRNAs to LINC01134, and five microRNAs were suggested to bind LINC01134 and were used for further validation after the intersection of the results from two databases (Fig. 4C). Based on the results from RNA pull-down experiments, miR-140-3p was mostly enriched in the LINC01134 group (Fig. 4D). Correlation analysis showed a significant negative correlation between LINC01134 and miR-140-3p (Fig. 4E). In addition, the overexpression of miR-140-3p significantly decreased the luciferase activity of LINC01134 WT, while it had no significant effects on the luciferase activity of LINC01134 MUT (Fig. 4F). The results of RIP also showed that LINC01134 and miR-140-3p were highly enriched in AGO2 precipitation (Fig. 4G). Results of the RNA pull-down assay showed that AGO2 and miR-140-3p were highly enriched in the biotin-labeled LINC01134 group (Fig. 4H). Finally, we validated the negative correlation between LINC01134 and miR-140-3p in BxPC-3 cells, MIA PaCa-2 cells, and PANC-1 cells using qRT-PCR (Fig. 4I).

**Fig. 5:**
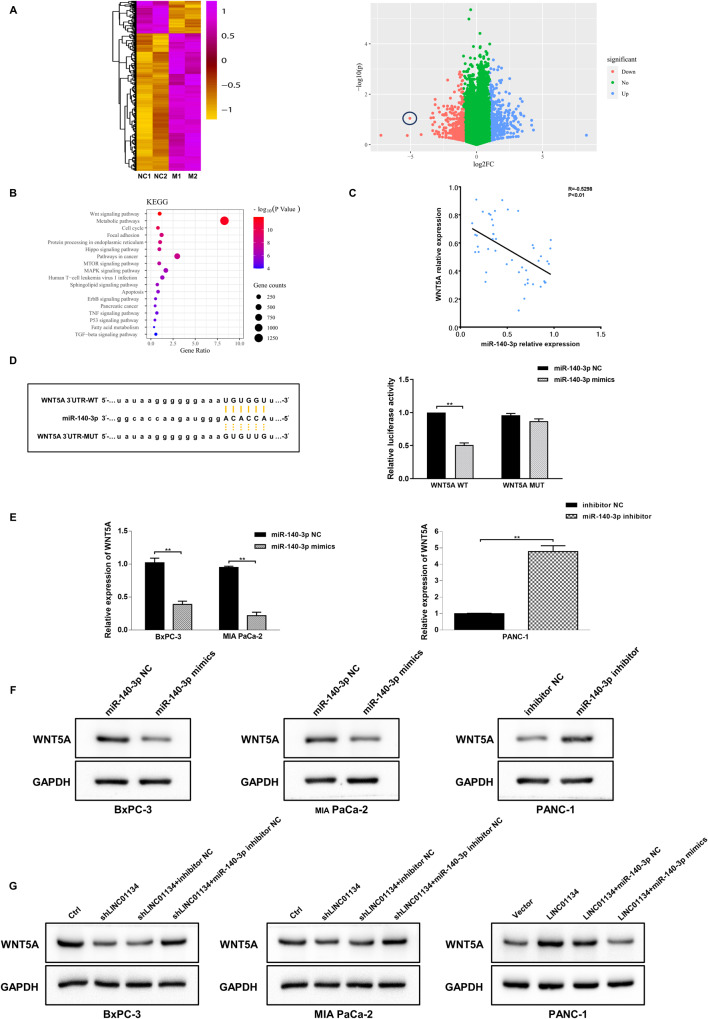
miR-140-3p targets WNT5A in PDAC. **A** Heatmap and volcano map showing differentially expressed genes in PDAC after the overexpression of miR-140-3p. **B** KEGG pathway analysis of the major pathways associated with differentially expressed genes. **C** Correlation analysis shows that miR-140-3p was negatively correlated with WNT5A. **D** Dual-luciferase reporter analysis detects the relative activity of luciferase in post-transfected PDAC cells. **E**, **F** Effects of silencing or overexpression of miR-140-3p on the expression of WNT5A in PDAC cells. **G** Effects of co-transfection of LINC01134 and miR-140-3p on the expression of WNT5A in PDAC cells. Data are expressed as mean, *p < 0.05, **p < 0.01.

To find the target downstream genes of miR-140-3p, we performed RNA sequencing analysis on the miR-140-3p overexpressing PDAC cells and identified a total of 1165 unique transcripts, including 217 upregulated and 948 down-regulated mRNAs, where WNT5A turned out to be the most representative one (Fig. 5A). Kyoto Encyclopedia of Genes and Genomes (KEGG) enrichment analysis indicated that the WNT pathway was the most enriched signaling pathway associated with these genes (Fig. 5B). Correlation analysis showed that the expression of miR-140-3p was correlated with WNT5A negatively (*p* < 0.01) (Fig. 5C). Figure 5D indicates that the overexpression of miR-140-3p significantly decreased the luciferase activity of WNT5A WT, while it had no significant effects on the WNT5A MUT. Meanwhile, the overexpression of miR-140-3p could significantly reduce the expression of WNT5A in BxPC-3 and MIA PaCa-2 cell lines, while silencing miR-140-3p increased the expression of WNT5A in PANC-1 cells (Fig. 5E, F). Further, we found that silencing LINC01134 also reduced the expression of WNT5A in BxPC-3 and MIA PaCa-2 cells, and this inhibitory effect could be reversed by silencing miR-140-3p. On the other hand, the overexpression of LINC01134 can increase the expression of WNT5A in PANC-1 cells, and the overexpression of miR-140-3p also reversed this effect (Fig. 5G). The above results suggest that LINC01134 exerts a biological function by regulating the expression of WNT5A in PDAC cells through competitively binding miR-140-3p.

The cell cycle analysis by flow cytometry indicated that silencing LINC01134 triggered the G1/S cell cycle arrest in BxPC-3 and MIA PaCa-2 cells, which is consistent with previous results, and this effect could be reversed by the overexpression of WNT5A. Similarly, the effect of LINC01134 overexpression on PANC-1 cells can be reversed by silencing WNT5A as shown in Fig. S4.

### LINC01134 affects the stem cell features and cell cycle of PDAC cells by regulating WNT5A, which in turn affects the drug resistance of PDAC

**Fig. 6:**
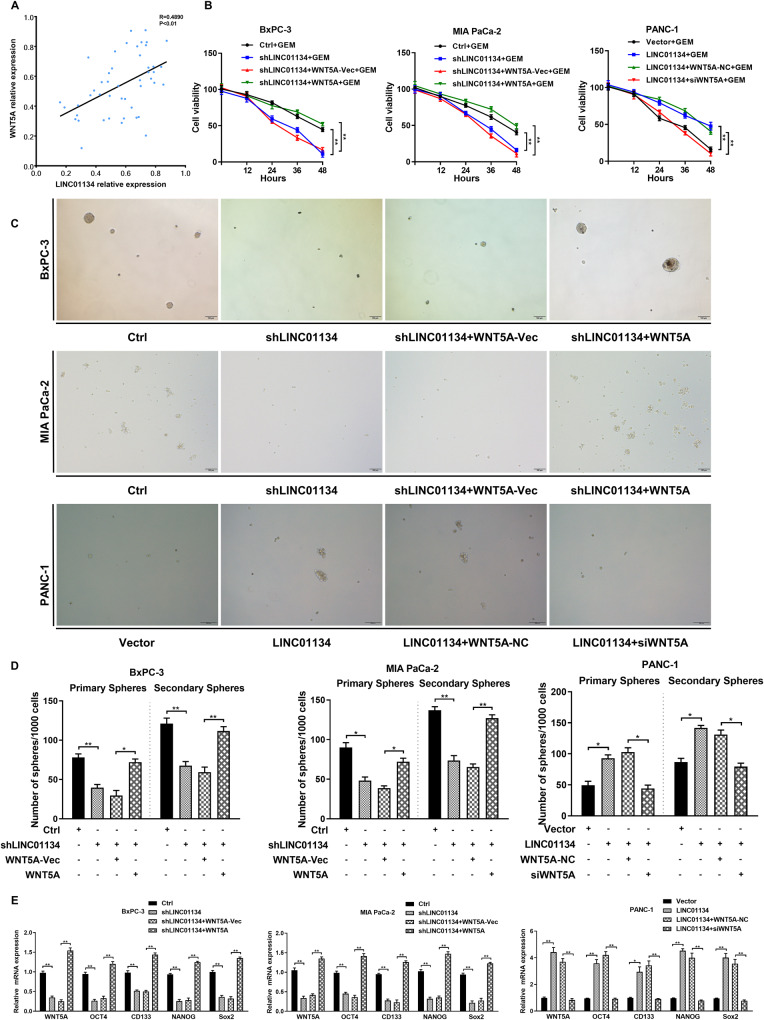
LINC01134 affects the stem cell features and cell cycle of PDAC cells by regulating WNT5. **A** Correlation analysis shows that the abundance of LINC01134 was positively correlated with WNT5A. **B** CCK-8 analysis of the effects of silencing or overexpressing LINC01134 and overexpressing or silencing WNT5A when co-transfected on the viability of PDAC cells. **C** Representative phase contrast images of tumorspheres formed by PDAC cells transduced with indicated constructs or a nontarget shRNA. **D** Statistical analysis of the primary and secondary sphere formation ability of PDAC cells as described in (**C**). **E** Effects of silencing or overexpression of LINC01134 and overexpression or silencing of WNT5A on the expression of the stemness-related genes when co-transfected. Data are expressed as mean, *p < 0.05, **p < 0.01.

We next investigated the link between LINC01134 and WNT5A. Correlation analysis showed a positive correlation between LINC01134 and WNT5A in PDAC tissues with a statistical significance (*p* < 0.01) (Fig. 6A). The results of CCK8 (Fig. 6B), sphere formation assay (Fig. 6C, D), and qRT-PCR (Fig. 6E) indicated that silencing LINC01134 enhanced the cytotoxic activity of GEM on BxPC-3 and MIA PaCa-2 cells and these effects can be reversed by the overexpression of WNT5A. In contrast, the overexpression of LINC01134 antagonized the cytotoxic activity of GEM on PANC-1 cells as well as increased the spheroid-forming ability and stem cell features of PANC-1 cells. These effects were also reversed by silencing WNT5A.

### LINC01134 can be a potential therapeutic target for PDAC resistance to GEM

**Fig. 7:**
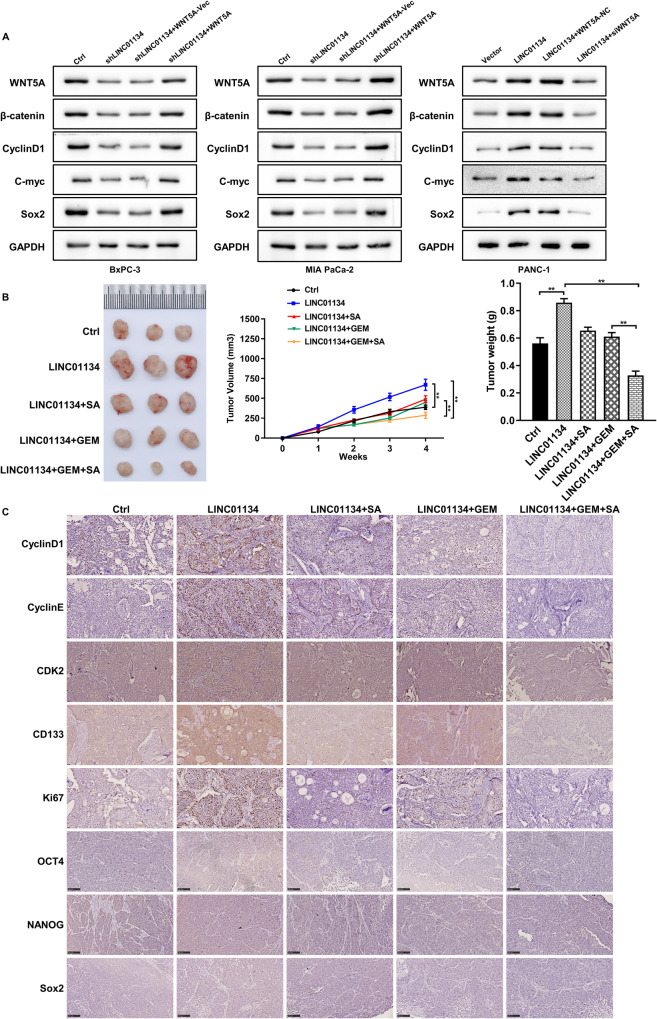
LINC01134 can be a potential therapeutic target for the treatment of PDAC resistance to GEM. **A** Silencing LINC01134 reduced the level of WNT5A and its downstream signaling activity in PDAC cells, and overexpression of LINC01134 enhanced the level of WNT5A and its downstream signaling activity in PDAC cells. **B** Overexpression of LINC01134 promoted the growth of transplanted tumors in PDAC nude mice and induced resistance to GEM. At the same time, the application of salinomycin effectively antagonized the pro-tumorigenic effect of LINC01134 and restored the sensitivity of transplanted tumors to GEM treatment. **C** IHC assays indicate the expression of Cyclin D1, Cyclin E, CDK2, CD133, Ki67, OCT4, NANOG and Sox2. Data are expressed as mean, *p < 0.05, **p < 0.01.

We next investigated the role of LINC01134 in regulating the WNT5A/WNT signaling pathway in pancreatic cancer. We found that silencing LINC01134 reduced the expression of WNT5A at the protein level and the downstream proteins, including β-catenin, Cyclin D1, C-myc, and Sox2. The effects can be reversed when WNT5A was overexpressed. In contrast, the overexpression of LINC01134 increased the expression of WNT5A and its downstream proteins, and this regulatory effect could also be reversed by silencing WNT5A (Fig. 7A). These results suggest that the LINC01134/WNT5A axis activates the WNT/β-catenin signaling pathway. To further investigate the function of LINC01134 in vivo, we established a nude mouse transplantation tumor model. It was observed that the LINC01134-overexpressed transplanted tumors grew significantly faster than the control group and gradually became resistant to GEM treatment over time. The application of salinomycin (a WNT/β-catenin pathway inhibitor) effectively antagonized the pro-tumorigenic effect of LINC01134 and restored the sensitivity of the transplanted tumors to GEM treatment (Fig. 7B). Subsequently, we performed immunohistochemical assays on the transplanted tumors and showed that the overexpression of LINC01134 increased the expression of Cyclin D1, Cyclin E, CDK2, CD133, Ki67, OCT4, NANOG and Sox2 at the protein level, while the combination of GEM and salinomycin can significantly inhibit the expression of these proteins (Fig. 7C). The above results suggest that LINC01134 could be a potential therapeutic target for PDAC resistance to GEM.

### FTO-mediated m6A modifications are involved in the upregulation of LINC01134

**Fig. 8:**
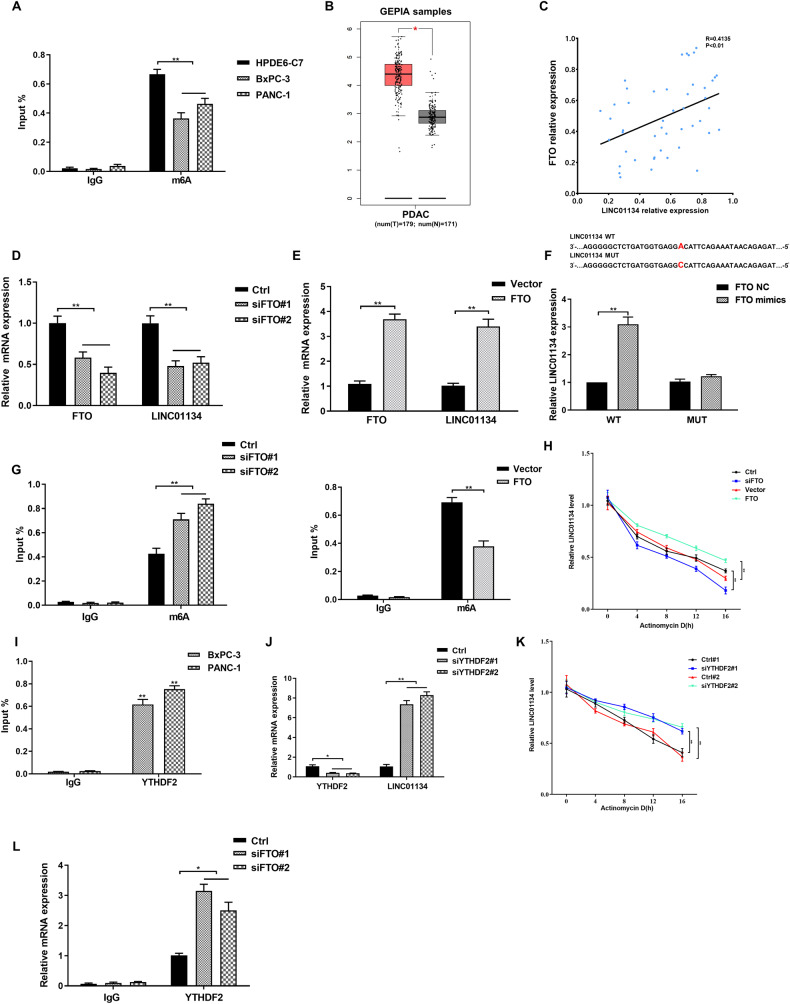
FTO-mediated m6A modifications are involved in the upregulation of LINC01134. **A** The results of MeRIP-qPCR indicate an overall lower level of m6A in BxPC-3 and PANC-1 cells than in HPDE6-C7 cells. **B** Analysis of the expression of FTO in PDAC using the GEPIA database. **C** Correlation analysis shows that the expression of FTO was significantly and positively correlated with the abundance of LINC01134. **D** Effects of silencing FTO on the expression of LINC01134. **E** Effects of overexpression of FTO on the expression of LINC01134. **F** An online engine (http://m6avar.renlab.org/) can be used to predict the m6A modification site of LINC01134 and the prediction was confirmed by a luciferase analysis report. **G** Effects of silencing or overexpression of FTO on the degree of m6A enrichment of LINC01134. **H** Effects of silencing or overexpression of FTO on the stability of LINC01134 in the presence of radio-luciferin D. **I** RIP assay confirms the binding between YTHDF2 and LINC01134. **J** Effects of silencing YTHDF2 on the expression of LINC01134. **K** Effects of silencing YTHDF2 on the stability of LINC01134 in the presence of radio-labeled D. **L** RIP experiments confirm the effects of silencing FTO on the expression of YTHDF2. Data are expressed as mean, *p < 0.05, **p < 0.01.

Many studies have found that N6-methyladenosine (m6A) modification can regulate the stability of RNA [32]. We used RMBase (http://rna.sysu.edu.cn/rmbase/index.php) and found that LINC01134 has many m6A modification sites. Therefore, we suspect that the high expression of LINC01134 in PDAC could be related to its m6A modification. Results of MeRIP-qPCR showed that the m6A levels of LINC01134 in pancreatic cancer cell lines (BxPC-3, PANC-1) were significantly lower than those in normal pancreatic cell lines (HPDE6-C7) (*p* < 0.01) (Fig. 8A). In addition, we found that FTO(Obesity-associated protein), a demethylase of m6A modification that induces RNA demethylation, is highly expressed in pancreatic cancer (Fig. 8B) [33]. Correlation analysis showed that the expression level of LINC01134 in pancreatic cancer was positively correlated with the abundance of FTO (Fig. 8C). Taken together, we hypothesized that FTO-mediated m6A modification was involved in the overexpression of LINC01134 in PDAC. Further studies were conducted to test this hypothesis.

After silencing FTO, a significant decrease in the expression of LINC01134 was observed (Fig. 8D), while an increased level of LINC01134 was observed upon the overexpression of FTO (Fig. 8E). Later, we used the online website (http://m6avar.renlab.org/) to predict the m6A modification sites of LINC01134, It was observed that the overexpression of FTO significantly increased the luciferase activity of LINC01134 WT, while it had no significant effects on the luciferase activity of LINC01134 MUT (Fig. 8F). The MeRIP-qPCR results showed that silencing of FTO increased the m6A modification of LINC01134 in PDAC cells, while the overexpression of FTO resulted in a decreased m6A modification of LINC01134 in PDAC cells (Fig. 8G). In the presence of radiotracer D, a drug that inhibits the RNA synthesis, we found that silencing FTO significantly reduced the stability of LINC01134, while the overexpression of FTO had the opposite effects (Fig. 8H). Taken together, FTO, a demethylase of m6A modification, may mediate the high expression of LINC01134 in PDAC.

In addition, the YT521-B homolog (YTH) structural domain family is an important “reader” protein in m6A modification [34]. Among all of them, YTH structural domain family protein 2 (YTHDF2) has been identified as an m6A reader that can accelerate the degradation of m6A modified RNAs [35]. RIP analysis confirmed that YTHDF2 can directly bind to LINC01134 (Fig. 8I). We also found that the level of LINC01134 was significantly increased when YTHDF2 was silenced (Fig. 8J). In addition, the stability of LINC01134 was significantly increased upon silencing YTHDF2 as shown in the radiolabel D analysis (Fig. 8K). RIP analysis also found that silencing FTO can significantly upregulate the expression of YTHDF2 at the mRNA level (Fig. 8L).

All of the results above indicate that m6A modification mediated by the demethylase FTO and the reader YTHDF2 plays an important role in maintaining the stability of LINC01134 in pancreatic cancer cells as well as relates to the high expression of LINC01134.

## Discussion

GEM is currently the first-line agent for the treatment of such advanced or metastatic pancreatic cancer [36]. The clinical benefit rate (CBR) of GEM in pancreatic cancer has reached 23.8% [36], but the good CBR has not translated into a satisfactory survival rate. The main reason for the low survival rate is the emergence of drug resistance [37]. Due to the lack of effective biological markers, GEM-resistant PDAC patients are often overlooked in clinical treatment. Therefore, there is an urgent need to find a reliable biomarker to maximize the efficacy of GEM. In this study, we identified LINC01134/miR-140-3p/WNT5A/WNT pathway as a critical axis regulating the resistance to GEM in PDAC and explored the role of LINC01134 in PDAC and its value for the prognostic assessment of GEM resistance for the first time.

There is growing evidence that lncRNAs play an important role in chem-resistance in malignancies. For example, LncRNA NR2F1-AS1 is associatedwith the oxaliplatin resistance in hepatocellular carcinoma [38]; lncARSR can promote the resistance to sunitinib in renal cell carcinoma [39]; LncRNA NORAD accelerates the progression of neuroblastoma and resistance to adriamycin through upregulation of HDAC8 [40]; lncRNA-HCP5 accelerates the progression of neuroblastoma and the resistance to adriamycin through miR-3619- 5p/AMPK/PGCAα/CEBPB axis which drives fatty acid oxidation, which in turn promotes stem cell features and the resistance to FOLFOX regimen in gastric cancer [41]. Many studies have identified mechanisms that lncRNAs are involved in the chemotherapy resistance in malignant tumors, including altering the efflux of chemotherapeutic agents, affecting the repair of damaged DNA, influencing the apoptosis of cells, and inducing mutations in drug targets [42]. In this study, we constructed a GEM-resistant PDX model to investigate the mechanism of PDAC resistance to GEM, and combined the pharmacological effects of GEM cytotoxic activity and the RNA sequencing results of the PDX model. We found that LINC01134 was highly expressed in the GEM-resistant PDX model and was closely associated with the G1/S-specific cyclins and cell cycle proteins-dependent LINC01134, a newly identified lncRNA. LINC01134 was found to recruit the transcription factor SP1 to the p62 promoter in hepatocellular carcinoma, which in turn activates the antioxidant pathway of p62 and regulates the resistance to oxaliplatin by affecting cell viability, apoptosis, and mitochondrial homeostasis [21]. Additional studies found that the transcription factor YY1 (yin yang 1) together with LINC01134, miR-324-5p, and IGF2BP1 (Insulin-like growth factor 2 mRNA binding protein 1), could form a positive feedback loop that mediates the progression of hepatocellular carcinoma [22]. Wang et al. showed that LINC01134 could activate AKT1S1, which further activates the NF-κB pathway to promote the metastasis of hepatocellular carcinoma [20]. Zheng and others’ study found that LINC01134 could act as a molecular sponge of microRNA-4784 to accelerate the progression of hepatocellular carcinoma [19]. LINC01134 has been studied only in hepatocellular carcinoma and its role in PDAC is unclear. In this study, we found that LINC01134 promotes the stem cell features and proliferation of PDAC cells by facilitating their transformation from the G0/G1 phase to the S phase. This mechanism can antagonize the cytotoxic activity of GEM and therefore, result in the resistance to GEM in PDAC cells.

The WNT/β-catenin signaling pathway is a highly conserved pathway whose aberrant activation plays an important role in the chemo-resistance in malignant tumors. FZD1 (Frizzled Class Receptor 1) has been found to mediate the multi-drug resistance in breast cancer by regulating the WNT/β-catenin signaling pathway [43]. lncRNA CRNDE, a molecular sponge of miR-181a-5p, is involved in regulating the expression of T cell factor 4 (TCF4)and β-catenin and regulates the proliferation and chemo-resistance in colorectal cancer by affecting the WNT/β-catenin signaling pathway [44]. The deubiquitinating enzyme USP20 (ubiquitin specific peptidase 20) regulates the deubiquitination of β-catenin and controls its stability, and as a result, positively regulates the tumorigenesis and chemoresistance in which cancer [45]. MASTL(microtubule associated serine/threonine kinase-like)promotes the progression and chemo-resistance of colon cancer by activating the WNT/β-catenin signaling pathway [46]. Salinomycin belongs to the potassium ion carrier antibiotics and is a potent inhibitor of the WNT/β-catenin signaling pathway [47]. The WNT/β-catenin signaling pathway is activated when WNT molecules bind to a co-receptor consisting of the Fzd receptor and lipoprotein receptor-related protein 5 or 6 (LRP5/6) and therefore, forming a WNT/Fzd/LRP ternary complex on the cell surface. Salinomycin can act on the WNT/Fzd/LRP complex to block the WNT-induced LRP6 phosphorylation and lead to the degradation of LRP6 protein, which inhibits the activation of the WNT/β-catenin signaling pathway [48–50]. In addition, salinomycin has been found to inhibit the expression of Cyclin D1 in prostate and breast cancers [49]. In this study, we found that LINC01134 can regulate the WNT/β-catenin signaling pathway by competitively binding miR-140-3p and affecting its targeting of WNT5A. The application of salinomycin can inhibit the expression of proteins upstream of the WNT/β-catenin signaling pathway. On the other hand, salinomycin can inhibit the expression of Cyclin D1 and therefore, reverse the drug resistance of PDAC cells to GEM.

m^6^A RNA methylation, a reversible epigenetic modification that differentially exists on mRNAs as well as ncRNAs, play a role in tumorigenesis and progression of multiple human malignancies [51]. Previous studies have shown that loss of m^6^A methylation modulates LncRNA expression levels [52–55]. So we wonder if LINC01134 is regulated by m^6^A PDAC. We found that the main reason that was responsible for the high expression of LINC01134 in PDAC was the level of m^6^A modification in LINC01134 that related to the expression of FTO and YTHDF2. In this study, we confirmed that FTO could induce the demethylation of LINC01134 in PDAC cells, and the high level of FTO led to a decreased m^6^A level of LINC01134. Since YTHDF2 mediates the degradation of m^6^A-modified RNA, we also found that the m^6^A of LINC01134 can be read by YTHDF2, which resulted in the reduction of m^6^A modification in LINC01134 m^6^A and led to a reduction in LINC01134 degradation. As a result, the stability of LINC01134 increased and its expression in PDAC increased.

In conclusion, we identified a novel regulatory mechanism in PDAC: the LINC01134/miR-140-3p/WNT5A/WNT pathway forms a new critical axis that antagonizes the cytotoxic activity of GEM by regulating the stem cell features and cell cycle of PDAC cells, leading to the development of resistance to GEM in PDAC. We also showed that the application of salinomycin could reverse this resistance mechanism by inhibiting upstream signaling of the WNT/β-catenin signaling pathway and suppressing the expression of Cyclin D1. These results identified the new prognostic markers and therapeutic targets for PDAC patients.

## Materials and methods

### Cell lines and cell culture

The standard human pancreatic cell line HPDE6-C7 and human PDAC cell line CaPan-1 were purchased from the American Tissue Culture Colection (ATCC), and the other human PDAC cell lines PANC-1, MIA PaCa-2, BxPC-3, SW1990, CFPAC-1, and AsPC-1 were purchased from the Cell Bank of the Typical Culture Collection Committee of the Chinese Academy of Sciences (Shanghai, China). Cells were cultured in an appropriate media containing 10% fetal bovine serum (FBS) (Gibco, city, State, USA). All standard growth mediums were supplemented with penicillin (100 U/mL) and streptomycin (100 μg/mL) to minimize the possibility of bacterial contamination. All cell lines were incubated at 37 °C in 100% humidity in an incubator containing 5% CO_2_/95% air.

### Chemical reagents and antibodies

Lipofectamine 2000 transfection reagent and total RNA extractant (TRIzol) were purchased from Invitrogen (Grand Island, NY, USA). The DAB substrate kits were purchased from Vector Laboratories, Inc. (Burlingame, CA, USA). Salinomycin、Gemcitabine were purchased from Selleck Chemicals (Houston, TX, USA). All other chemical reagents were purchased from Sigma-Aldrich (St. Louis, MO, USA) unless otherwise stated.

### Patients and specimens

All studies involving human samples were reviewed and approved by the Ethics Committee of the Second Hospital of Jilin University, and the study protocols complied with the ethical standards of the Declaration of Helsinki. Pancreatic cancer tissues and paracancerous tissues were obtained from PDAC patients in the Second Hospital of Jilin University (*n* = 70). Clinicopathological features examined included age, gender, history of smoking and alcohol consumption, tumor size, tumor stage, lymph node metastasis, and distant metastasis. Tumor staging was based on the 6th edition of the International Union Against Cancer tumor-lymph node-metastasis (TNM) classification.

### Quantitative real-time polymerase chain reaction (qRT-PCR)

Total RNA was extracted from selected cells or tissues using TRIzol reagent (Invitrogen, Carlsbad, CA, USA) according to instructions provided by the manufacturer. Complementary DNA (cDNA) was synthesized using RevertAid First Strand cDNA Synthesis Kit (Thermo Scientific, #K1622, city, state, USA) and poly(A) Polymerase Reaction Buffer (NEB, M0276s, city, state, USA) to synthesize complementary DNA (cDNA). Real-time qRT-PCR analysis was performed using the Platinum SYBR Green qPCR SuperMix-UDG kit (Life Technologies, Gaithersburg, MD, USA) according to the instructions provided by the manufacturer. Nucleus/Mass Separation Kit (BioVision, San Francisco, CA, USA) was used to separate RNA from the cytoplasm and nucleus according to the protocols provided by the manufacturer. The 2-ΔΔCt method was used to quantify the ploidy changes in RNA expression. The detailed primer sequences used in this study are listed in Table S2.

### Cell cycle assay

Cells were inoculated in six-well plates for 48 h. Cells were collected and the supernatant was discarded after centrifugation at 1000 rpm for 5 min. Cell pellets were washed twice with phosphate buffer (PBS), and the supernatant was discarded by another centrifugation. Later, cells were incubated with 1 mL of DNA staining solution and 10 μL of permeabilization solution for 30 min at room temperature in dark, and the cell cycle distribution was analyzed using flow cytometry (Beckman Coulter, CA, USA).

### Western blot

Cells or tissues were homogenized and lysed using lysis buffer, and total protein concentration was determined by the BCA method (Beyotime, Shanghai, China). β-mercaptoethanol, and bromophenol blue were added to the sample buffer for electrophoresis. Proteins were separated through 10% polyacrylamide gel electrophoresis and then transferred to polyvinylidene difluoride membranes (Bio-Rad, Shanghai, China). Membranes were incubated with primary antibodies at the optimized dilution factor overnight at 4 °C and later a 2-h incubation with the secondary antibody at room temperature. The reaction bands were visualized using an enhanced chemiluminescence system, and the intensity of bands was quantified using an image analysis system.

### Sphere forming experiment

We inoculated 500 cells/well in an ultra-low adsorption six-well plate (Corning, NY, USA) in 5 mL of DMEM/F12 medium (Invitrogen, Shanghai, China) supplemented with B27 (1:50) (Gibco, Shanghai, China), 20 ng/mL EGF (Sigma, Shanghai, China), and 20 ng/mL bFGF (Sigma, Shanghai, China). Cells were incubated for 7 days in the same cell culture condition as described in section 2.1 (primary spheroid formation), digested with trypsin, centrifuged and resuspended, and then incubated for another 7 days using the same procedure to generate secondary spheroid. The number of spheroids formed was counted under a microscope.

### Transfection of shRNAs and siRNAs

Short hairpin RNAs(shRNAs) specifically targeting LINC01134, small interfering RNAs (siRNAs) explicitly targeting miR-140-3p, WNT5A, FTO, YTHDF2, and overexpression plasmids of LINC01134, miR-140-3p, and WNT5A were purchased from GenePharma (Shanghai, China). These shRNAs, siRNAs, and overexpression plasmids were transfected into PDAC cells using Lipofectamine 2000 according to the protocols provided by the manufacturer. In in vivo experiments, shLINC01134 was transfected into PDAC cells using lentivirus.

### Cell proliferation assay and colony formation assay

Transfected cells (1 × 10^3^/well) were inoculated in 96-well plates in complete growth media containing GEM at different experimental doses. The number of cells was counted every 12 h using a cell counting kit (CCK-8, Dojindo, Japan), and the absorbance values were measured at 450 nm using an enzyme marker (Spectramax plus384, Molecular Devices, USA). A total of 4 assays were performed as replicates. In the colony formation assay, transfected cells (1 × 10^3^/well) were inoculated in 6-well plates and cultured with or without GEM for 10–14 days. After washing 2 times with PBS, the cell colonies were counted after fixing with 70% methanol for 30 min and staining with 0.5% crystal violet for 30 min.

### Immunohistochemistry (IHC)

Tissue sections were pretreated in a desiccator at 60 °C for 1 h, dewaxed in xylene, and hydrated using graded ethanol for IHC experiments using thermally mediated antigen repair citrate (0.01 M, pH 6.0). Endogenous peroxidase activity was blocked for 15 min at room temperature with 3% H_2_O_2_. The primary antibody was incubated with 5% bovine serum albumin (BSA) (Beijing InnoChem Science & Technology Co., Ltd, Beijing, China) for 1 h to close the non-specific binding site and at then 4 °C overnight. The cells were rewarmed for 30 min, rinsed three times with PBS (5 min each), then incubated with a horseradish peroxidase-labeled secondary antibody for 1 h. The HRP DAB kit (Thermo Scientific, Shanghai, China) was used for color development, and the nuclei were re-stained with hematoxylin and sealed with neutral gel. Images were taken using an Olympus X71 inverted microscope (Olympus Corp., Tokyo, Japan).

### Dual-luciferase reporter assay

The complementary DNA fragment of the 3′ untranslated region (UTR) of the wild-type or mutant LINC01134 fragment was subcloned downstream of luciferase in the pGL3-Basic luciferase reporter gene (Promega, Beijing, China). The firefly luciferase gene containing wild-type or mutant LINC01134 fragment was cotransfected with an empty vector or the miR-140-3p mimic. After 48 h post-transfection, luciferase activity was measured using the Dual-Luciferase Kit (Promega, Beijing, China). The binding between WNT5A and miR-140-3p was validated using the same method.

### Fluorescence in situ hybridization

Fluorescence in situ hybridization (FISH) was used to determine the subcellular localization of LINC01134. Cells were fixed with 4% paraformaldehyde at room temperature, treated with 0.5% Triton X-100 for 5 min at 4 °C, and incubated with pre-hybridization buffer for 30 min at 37 °C. Cells were treated with the LINC01134 probe (RiboBio, Guangzhou, China) overnight in dark. Cells were washed sequentially using different wash buffers in dark at room temperature according to the instructions provided by the manufacturer. Finally, the nuclei were re-stained using DAPI, observed, and photographed using fluorescence microscopy (Olympus Corp., Tokyo, Japan).

### Methylated RNA immunoprecipitation qPCR (MeRIP-qPCR)

Total intracellular RNA was extracted using the TRIzol reagent. The anti-m6A antibody or immunoglobulin G (IgG) antibody (Cell Signaling Technology, Danvers, MA, USA) (3 μg) was conjugated to protein A/G magnetic beads and mixed with 100 μg aliquots of total RNA in IP buffer containing RNase/protease inhibitors. The m6A-modified RNA was eluted with 6.7 mM of N6-methyladenosine 5′-monophosphate sodium salt for 2 elutions at 4 °C for 1 h. qRT-PCR analysis was subsequently performed to determine the abundance of m6A enrichment on LINC01134.

### RNA immunoprecipitation (RIP) test

Using the Magna RIPTM RNA-binding protein immunoprecipitation kit (Millipore, USA) according to the instructions provided by the manufacturer, cell extracts were briefly immunoprecipitated with antibodies against AGO2 or IgG on magnetic beads at 4 °C for 6 h. The complexes were removed using 0.1% SDS/proteinase K (0.5 mg/mL, 55 °C, 30 min) and the beads were then washed using bead elution buffer. The immunoprecipitated proteins and RNAs were detected using Western blot and qRT-PCR, respectively.

### RNA pull-down experiment

An RNA pull-down analysis was performed using the Pierce Magnetic RNA-Protein Pull-Down Kit (Thermo Scientific, 20164) according to the instructions provided in the kit. In short, cell lysates were treated with RNAase-free DNAase I. The cell lysates after treatment were added to streptavidin-labeled magnetic beads with biotin-labeled LINC01134 probes and co-incubated at indoor temperature to capture proteins/miRNAs that may interact with LINC01134. Beads were washed with bead elution buffer, and then proteins and RNAs in the captured protein-RNA complexes were analyzed using Western blot and qRT-PCR, respectively.

### Establishment of the xenograft subcutaneous transplantation mouse model

Four-week-old BALB/c nude mice were purchased from the Experimental Animal Center of Jilin University (Changchun, China). All experimental animal protocols were performed following the National Institutes of Health Guide for the Use of Laboratory Animals. The animal experimental protocols for this study were reviewed and approved by the Animal Experimentation Ethics Committee of the First Hospital of Jilin University. BxPC-3 cells from the experimental group or negative control were injected subcutaneously at 2 × 10^6^ cells into the lateral abdominal region of the mice. The tumor volume was measured weekly and calculated as V = (length × width^2^)/2. Mice were executed four weeks later, and tumor weight and length were measured.

### Statistical analysis

All values were shown as mean ± standard deviation (SD). Comparisons between groups were made by *t*-test or one-way analysis of variance (ANOVA). Qualitative data were analyzed by Chi-square test. Linear regression analysis was performed to analyze the correlations between gene expression levels. Statistical analyses were performed using GraphPad Prism v8.0 (GraphPad, Inc., San Diego, CA, USA) and Statistical Software Package for Social Sciences (v 22.0; SPSS, Inc., Chicago, IL, USA). Differences were statistically significant at *p* < 0.05.

### Supplementary information

Supplementary Table 1

Supplementary Table 2

Supplementary Figure 1

Supplementary Figure 2

Supplementary Figure 3

Supplementary Figure 4

supplementary legends

Supplementary Material

checklist

## Data Availability

The datasets generated during and/or analysed during the current study are available in the [figshare] repository, [https://figshare.com/search?q=10.6084%2Fm9.figshare.24335371] and [https://figshare.com/search?q=10.6084%2Fm9.figshare.24175068].
