# Supplementary material for: FTO-mediated LINC01134 stabilization to promote chemoresistance through miR-140-3p/WNT5A/WNT pathway in PDAC
Source: Cell Death Dis. 2023 Nov 1;14(11):713. doi: 10.1038/s41419-023-06244-7 (PMC10620239; doi:10.1038/s41419-023-06244-7)
Supplement: Supplementary file 1 — Supplementary Table 1 [file 41419_2023_6244_MOESM1_ESM.docx]

**Table S1: Relationship between LINC01134 and clinicopathological parameters in 70 PDAC patients**

| Variables | All cases | LINC01134 expression | | *P* |
| --- | --- | --- | --- | --- |
|  |  | Low (n = 34) | High (n = 36) |  |
| **Age (years)** |  |  |  | 0.097 |
| <50 | 42 | 17 | 25 |  |
| ≥50 | 28 | 17 | 11 |  |
| **Gendar** |  |  |  | 0.871 |
| Male | 48 | 23 | 25 |  |
| Female | 22 | 11 | 11 |  |
| **Alcohol history** |  |  |  | 0.238 |
| No | 38 | 16 | 22 |  |
| Yes | 32 | 18 | 14 |  |
| **Smoking** |  |  |  | 0.348 |
| No | 21 | 12 | 9 |  |
| Yes | 49 | 22 | 27 |  |
| **Tumor size (cm)** |  |  |  | **0.017** |
| <4 | 33 | 21 | 12 |  |
| ≥4 | 37 | 13 | 24 |  |
| **TNM stage** |  |  |  | 0.241 |
| Ⅰ-Ⅱ | 30 | 17 | 13 |  |
| Ⅲ-Ⅳ | 40 | 17 | 23 |  |
| **Lymph node invasion** |  |  |  | **0.001** |
| Absent | 42 | 29 | 13 |  |
| Present | 28 | 5 | 23 |  |
| **Metastasis** |  |  |  | **0.03** |
| No | 48 | 29 | 19 |  |
| Yes | 22 | 5 | 17 |  |
