# Supplementary material for: FTO-mediated LINC01134 stabilization to promote chemoresistance through miR-140-3p/WNT5A/WNT pathway in PDAC
Source: Cell Death Dis. 2023 Nov 1;14(11):713. doi: 10.1038/s41419-023-06244-7 (PMC10620239; doi:10.1038/s41419-023-06244-7)
Supplement: Supplementary file 2 — Supplementary Table 2 [file 41419_2023_6244_MOESM2_ESM.docx]

**Table S2 The sequences of primers for RT-qPCR**

| cDNA | Primers | Sequences |
| --- | --- | --- |
| LINC01134 | forward | 5'-CATGTTTGAGCGAGGACCCC-3' |
|  | reverse | 5'-ATAGGAGAGGGTGGGCTTGA-3' |
| U6 | forward | 5'-CGCAAGGATGACACGCAAATTC-3' |
|  | reverse | 5'-GTGCAGGGTCCGAGGT-3' |
| β-actin | forward | 5'-CCTTCCTGGGCATGGAGTC-3' |
|  | reverse | 5'-TGATCTTCATTGTGCTGGGTG-3' |
| CD133 | forward | 5'-ACACTACCAAGGACAAGGCG-3' |
| OCT4  NANOG  SOX-2  FTO  miR-140-3p  WNT5A | reverse  forward  reverse  forward  reverse  forward  reverse  forward  reverse  forward  reverse  forward  reverse | 5'-TCTCCAACGCCTCTTTGGTC-3'  5'-CAAAGCAGAAACCCTCGTGC-3'  5'-AACCACACTCGGACCACATC-3'  5'-GATGCCTCACACGGAGACTG-3'  5'-TTGACCGGGACCTTGTCTTC-3'  5'-AGGATAAGTACACGCTGCCC-3'  5'-TTCATGTGCGCGTAACTGTC-3'  5'-AGAATGTCTGTGACGATGTGG-3'  5'-GCACTTTCTGTATCGATTGCC-3'  5'-TACCACAGGGTAGAACCACGG-3'  5'-CGAATTCTAGAGCTCGAGGCAGG-3'  5'-ACATCGACTATGGCTACCGC-3'  5'-AGGTTCATGAGGATGCGAGC-3' |
