# Supplementary figures and images for: FTO-mediated LINC01134 stabilization to promote chemoresistance through miR-140-3p/WNT5A/WNT pathway in PDAC
Source: Cell Death Dis. 2023 Nov 1;14(11):713. doi: 10.1038/s41419-023-06244-7 (PMC10620239; doi:10.1038/s41419-023-06244-7)

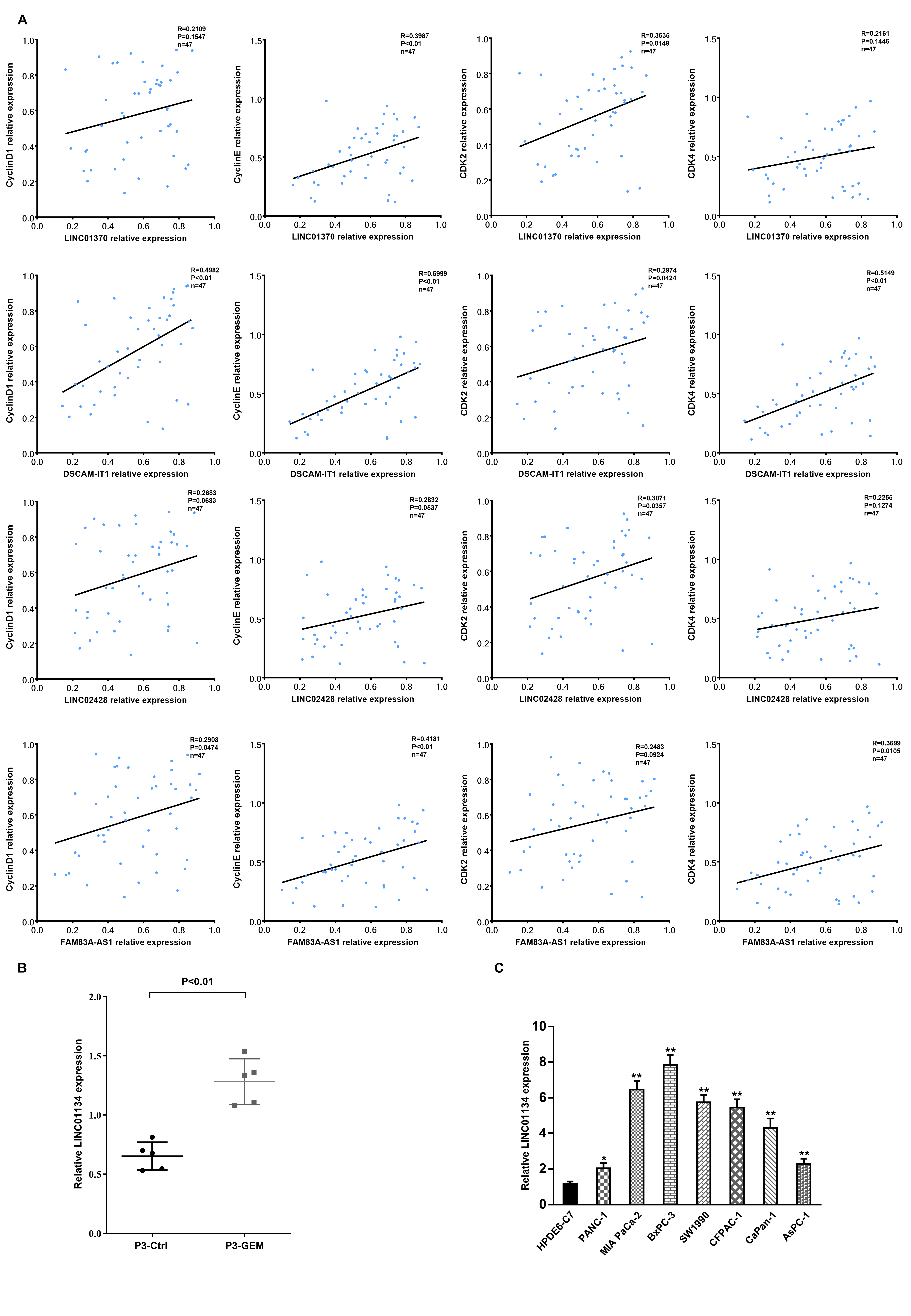

Supplement: Supplementary file 3 — Supplementary Figure 1 [file 41419_2023_6244_MOESM3_ESM.tif]

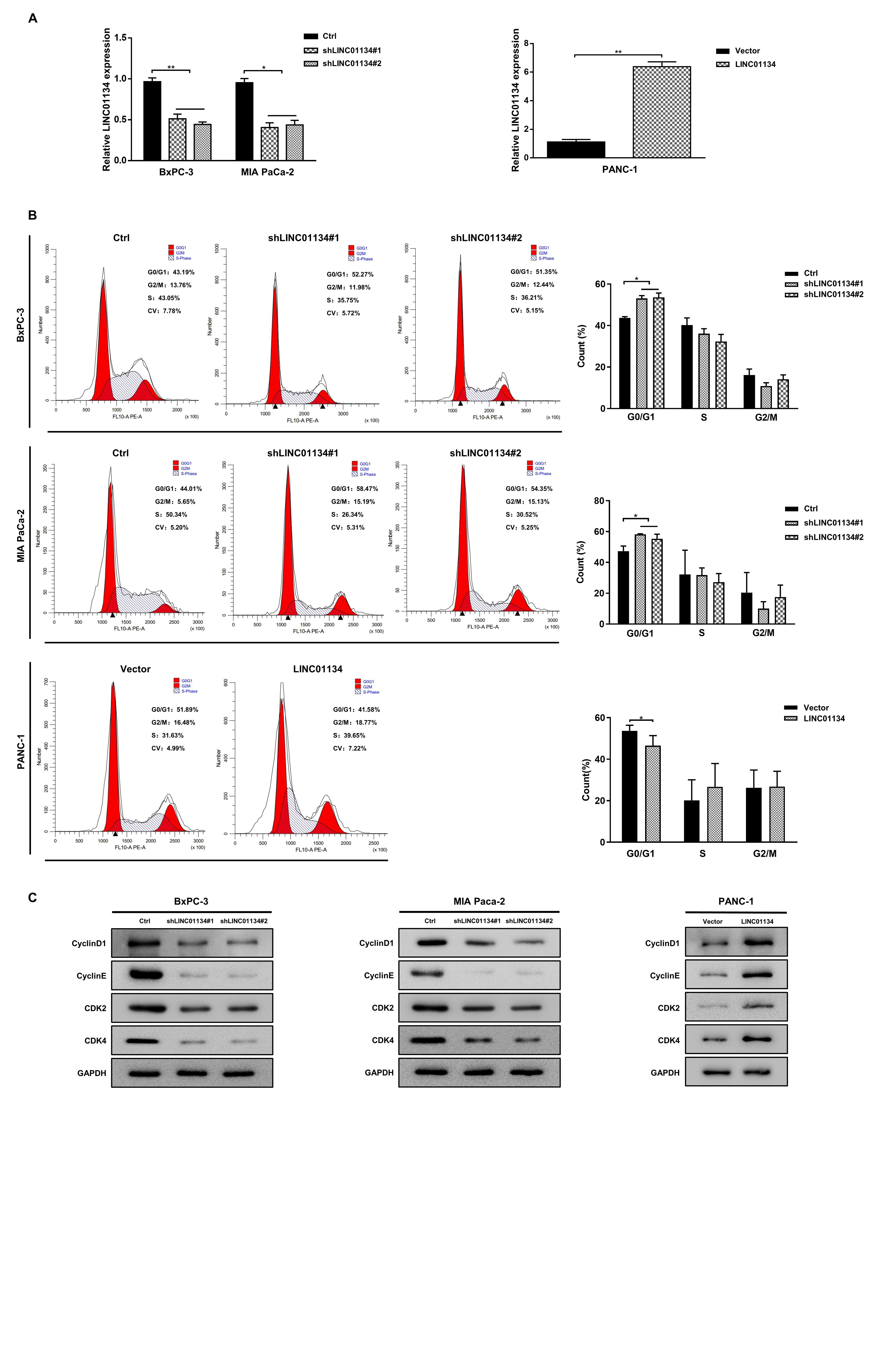

Supplement: Supplementary file 4 — Supplementary Figure 2 [file 41419_2023_6244_MOESM4_ESM.tif]

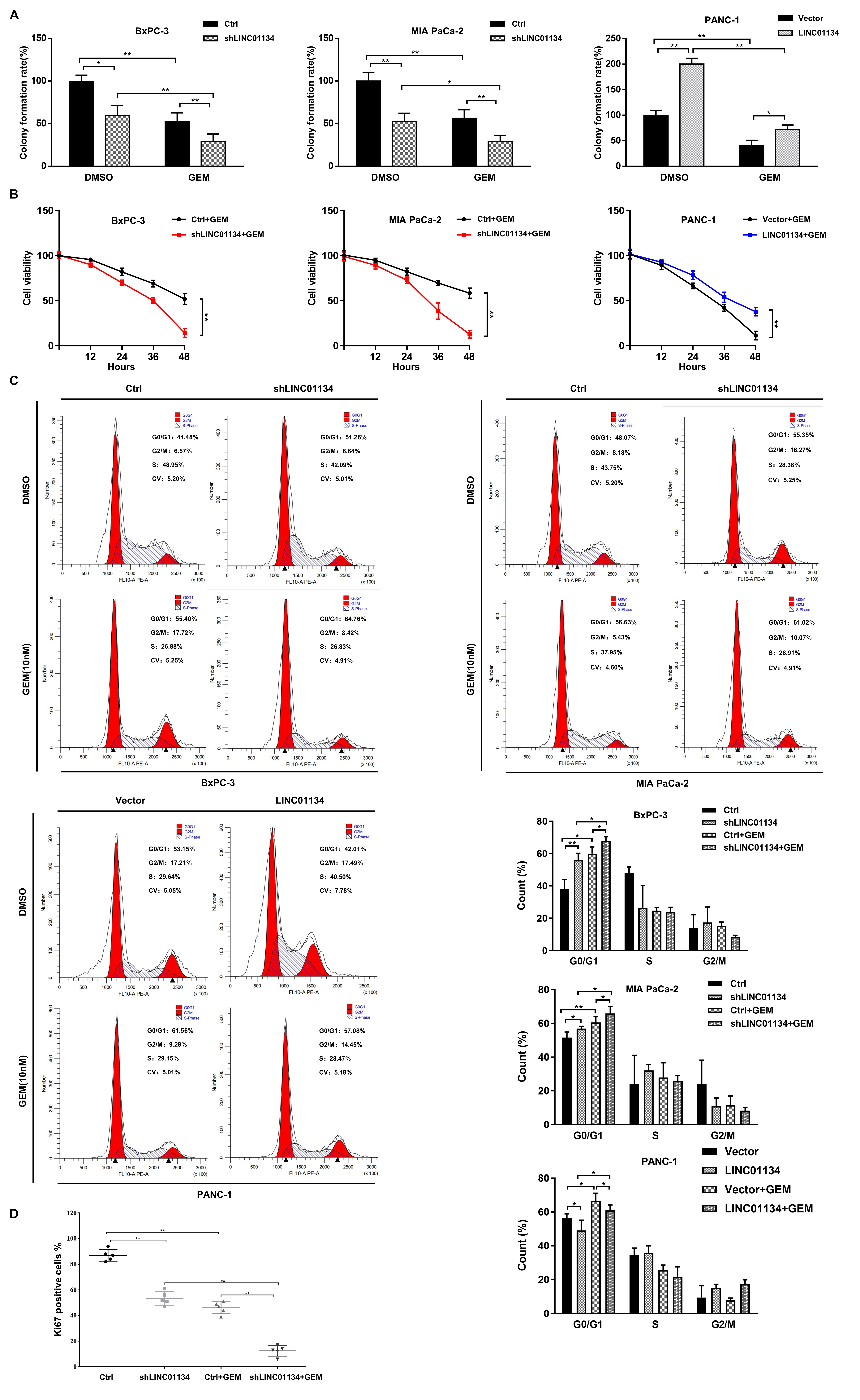

Supplement: Supplementary file 5 — Supplementary Figure 3 [file 41419_2023_6244_MOESM5_ESM.tif]

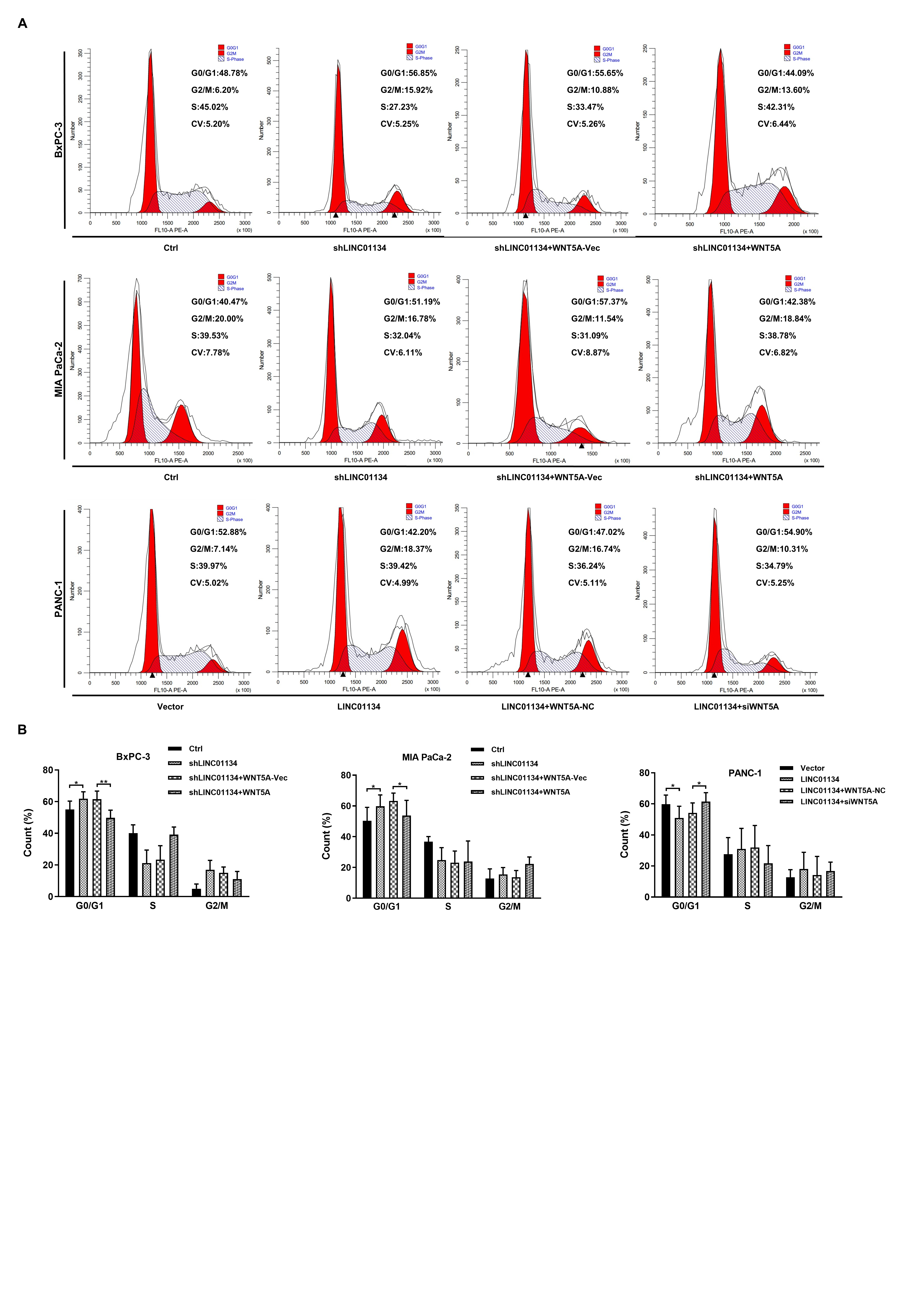

Supplement: Supplementary file 6 — Supplementary Figure 4 [file 41419_2023_6244_MOESM6_ESM.tif]
