# Supplementary material for: FTO-mediated LINC01134 stabilization to promote chemoresistance through miR-140-3p/WNT5A/WNT pathway in PDAC
Source: Cell Death Dis. 2023 Nov 1;14(11):713. doi: 10.1038/s41419-023-06244-7 (PMC10620239; doi:10.1038/s41419-023-06244-7)
Supplement: Supplementary file 7 — supplementary legends [file 41419_2023_6244_MOESM7_ESM.doc]

**Figure S1 related to Figure 1. LINC01134 is highly expressed in GEM-treated patient-derived xenograft (PDX) PDAC model.** (A) Correlation analysis of different lncRNAs with Cyclin D1, Cyclin E, CDK2, and CDK4. (B) Comparison of the expression of LINC01134 in 3 generations of PDX mice treated with saline or gemcitabine. (C) The expression of LINC01134 in normal pancreatic cell line and other pancreatic cancer cell lines. Data are expressed as mean, ^*^*p*<0.05, ^**^*p*<0.01.

**Figure S2 related to Figure 3. Silencing LINC01134 arrests the G1/S cell cycle and suppresses stem cell features in PDAC cells.** (A) qRT-PCR validates the transfection efficiency of LINC01134 down-regulation or overexpression. (B) Effects of silencing LINC01134 or overexpression of on the cell cycle of PDAC cells.Effects of overexpression of LINC01134 on the cell cycle in PDAC. (C) Western blot analysis to detect the effects of silencing or overexpression of LINC01134 on the expression of Cyclin D1, Cyclin E, CDK2, and CDK4.

**Figure S3 related to Figure 4. LINC01134 promotes chemotherapy resistance of PDAC to GEM in vitro and in vivo.** (A) Colony formation of cells silenced or overexpressing LINC01134 after GEM treatment. (B) CCK-8 assay indicates the changes of cell viability at different time points after GEM treatment to cells that had silenced or overexpressed LINC01134. (C) Cell cycle profiles of cells that had silenced or overexpressed LINC01134 after GEM treatment. (D) The expression of Ki67 in xenograft tumors formed from PDAC cells transfected with Ctrl or shLINC01134 in GEM-treated nude mice. Data are expressed as mean, ^*^*p*< 0.05, ^**^*p*< 0.01.

**Figure S4 related to Figure 5. LINC01134 affects the stem cell features and cell cycle of PDAC cells by regulating WNT5A.** (A) Effects of silencing or overexpression of LINC01134 and overexpression or silencing of WNT5A on the cell cycle of PDAC cells when co-transfected. (B) Effects of silencing or overexpression of LINC01134 and overexpression or silencing of WNT5A on the cell cycle of PDAC when co-transfected. Data are expressed as mean, ^*^*p*< 0.05, ^**^*p*< 0.01.
