# Supplementary material for: FTO-mediated LINC01134 stabilization to promote chemoresistance through miR-140-3p/WNT5A/WNT pathway in PDAC
Source: Cell Death Dis. 2023 Nov 1;14(11):713. doi: 10.1038/s41419-023-06244-7 (PMC10620239; doi:10.1038/s41419-023-06244-7)
Supplement: Supplementary file 8 — Supplementary Material [file 41419_2023_6244_MOESM8_ESM.docx]

**Figure 1**

**B**


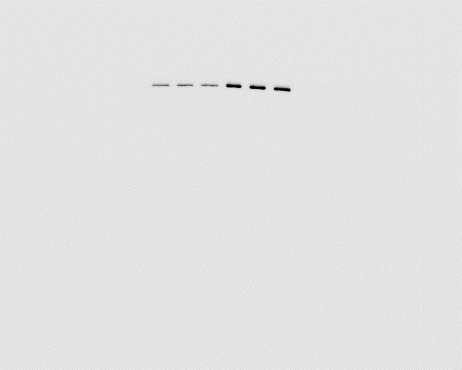

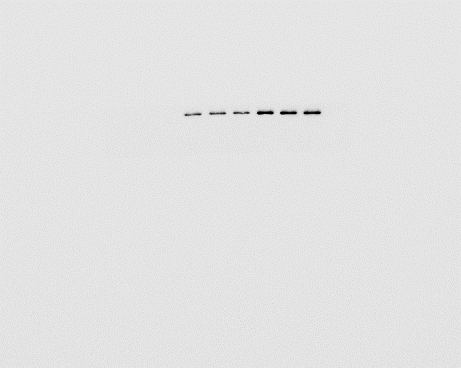


**Figure1-CyclinD1 Figure1-CyclinE**


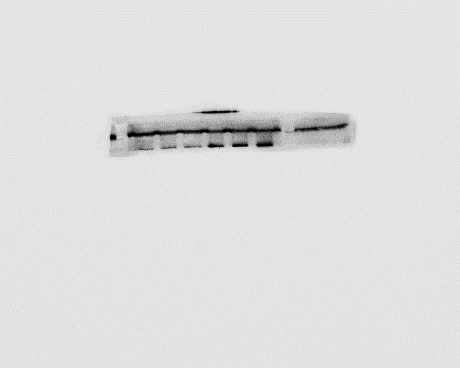

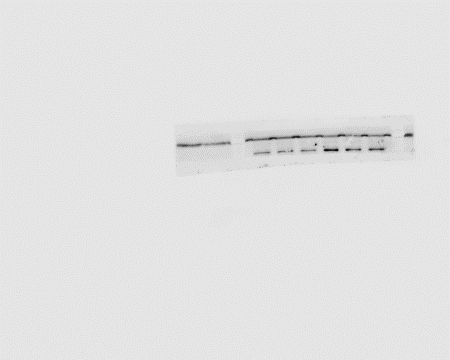


**Figure1-CDK2 Figure1-CDK4**


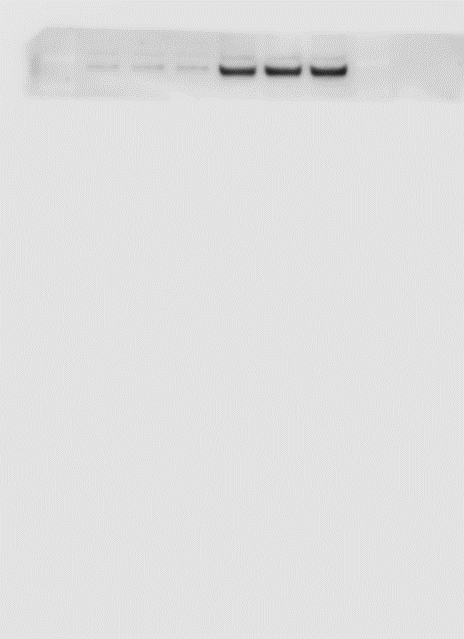

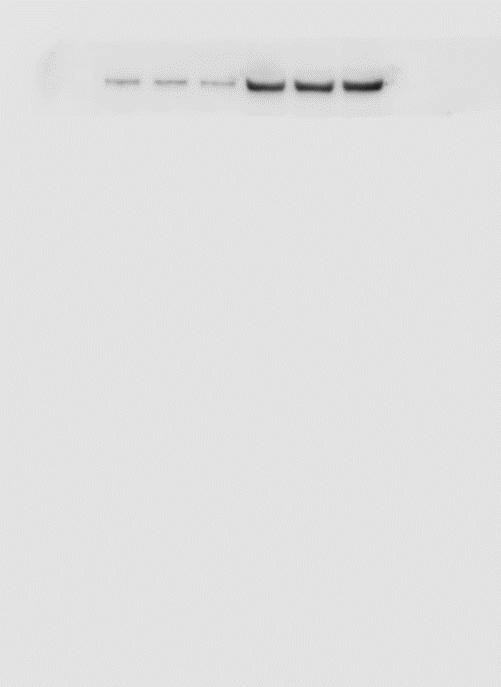


**Figure1-CD133 Figure1-OCT4**


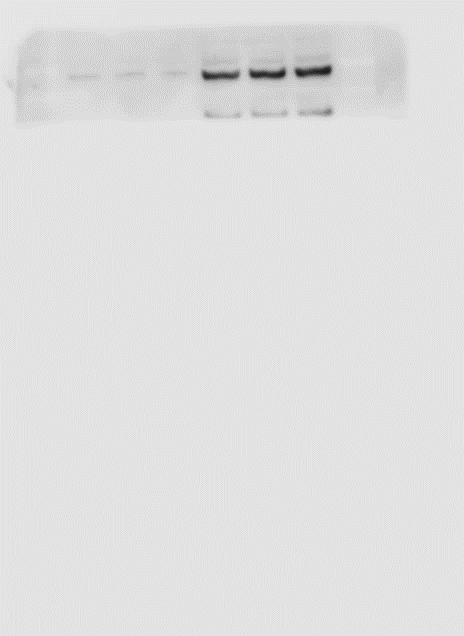

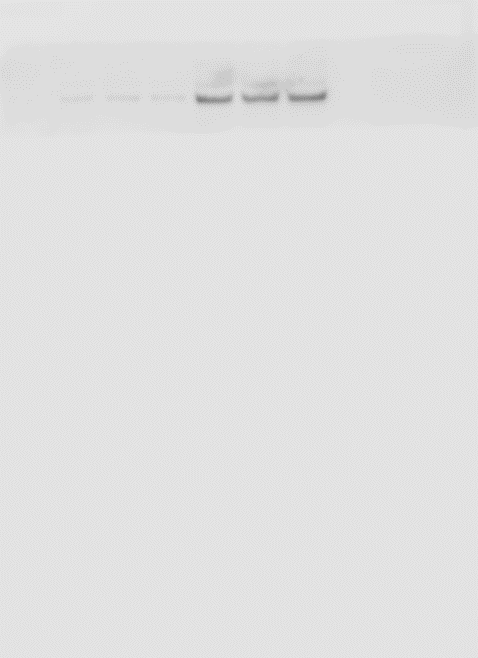


**Figure1-NANOG Figure1-Sox2**


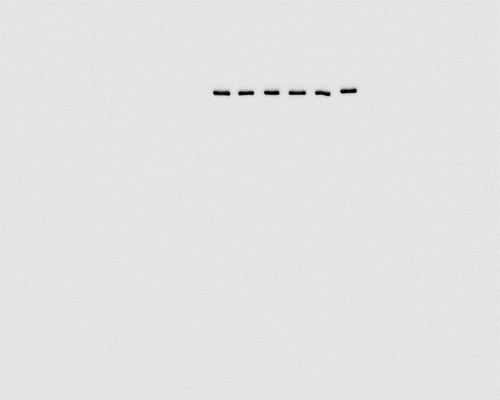


**Figure1-GAPDH**

**Figure 2**

**B**


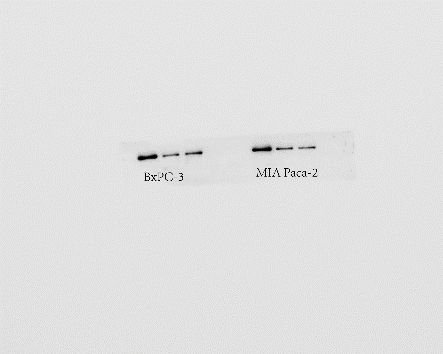

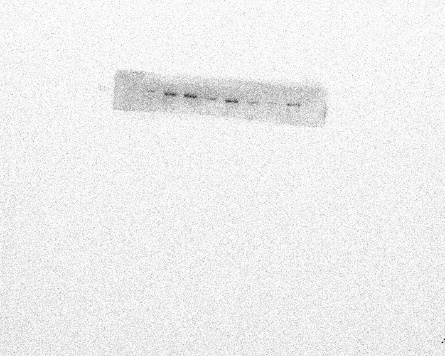


**Figure2-B-CD133 Figure2-B-CD133-PANC1**


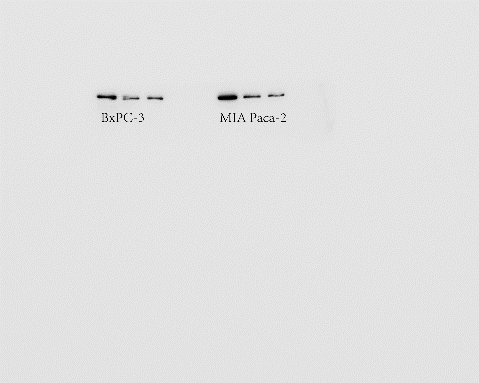

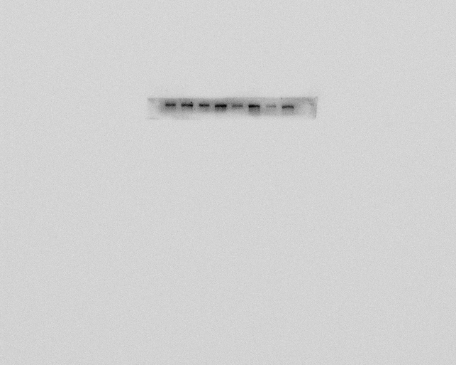


**Figure2-B-NANOG Figure2-B-NANOG-PANC1**


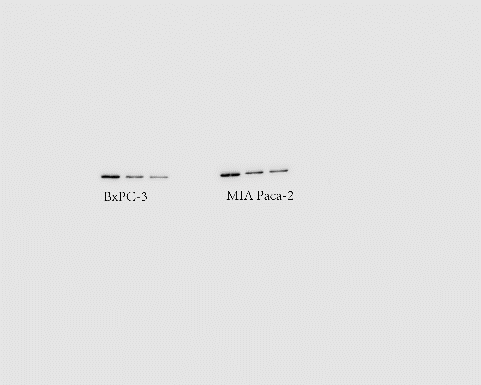

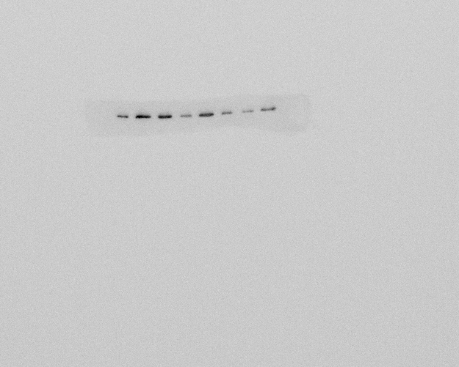


**Figure2-B-Sox2 Figure2-B-Sox2-PANC1**


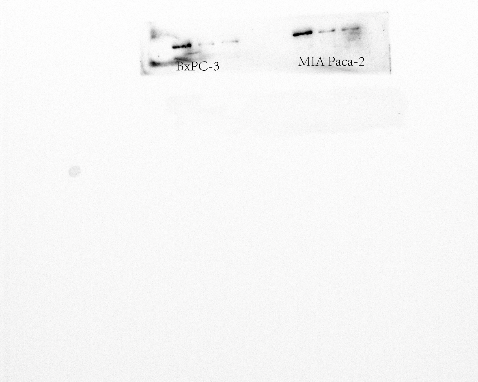

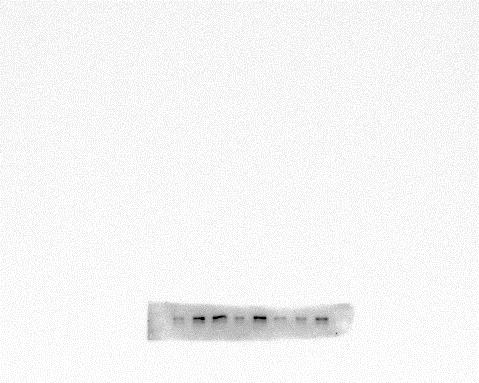


**Figure2-B-OCT4 Figure2-B-OCT4-PANC1**


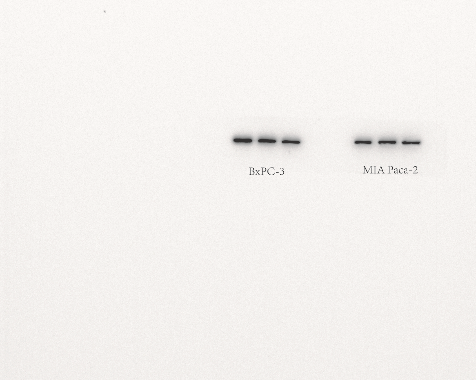

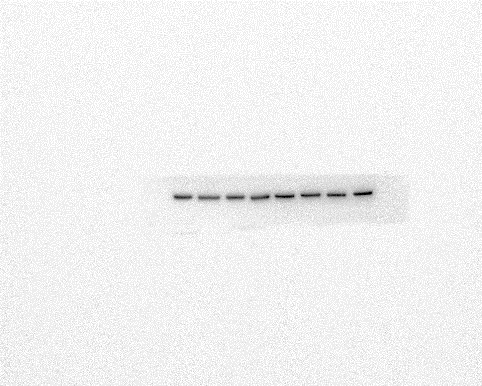


**Figure2-B-GAPDH Figure2-B-GAPDH-PANC1**

**Figure 3**

**E**


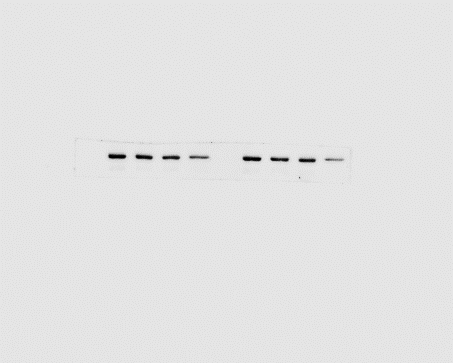

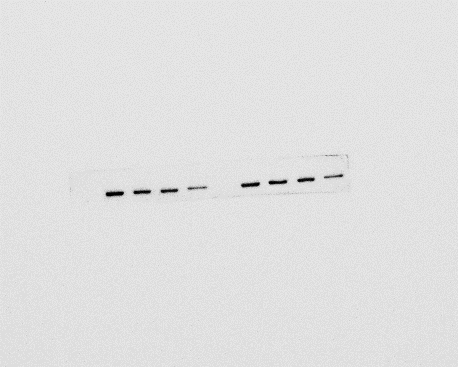


**Figure3 -cyc D1 Figure3 -cyc E**


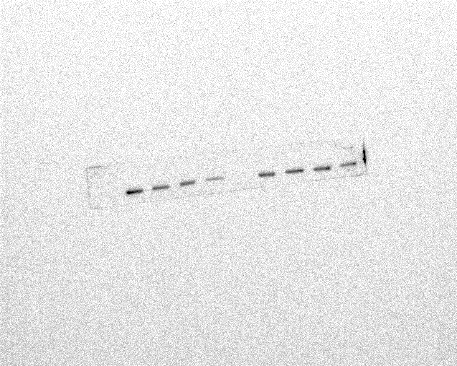

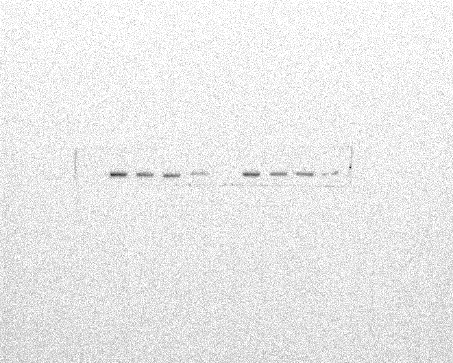


**Figure3 -CDK2 Figure3 -CDK4**


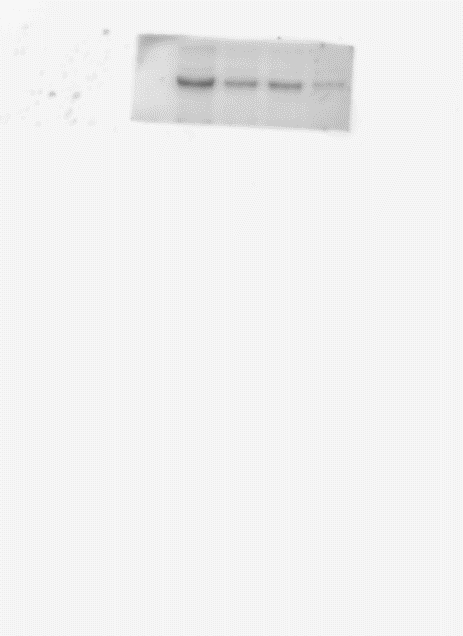

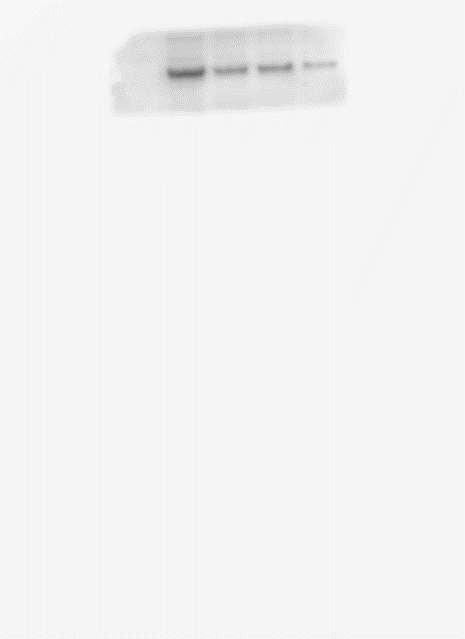


**Figure3-CD133 Figure3-OCT4**


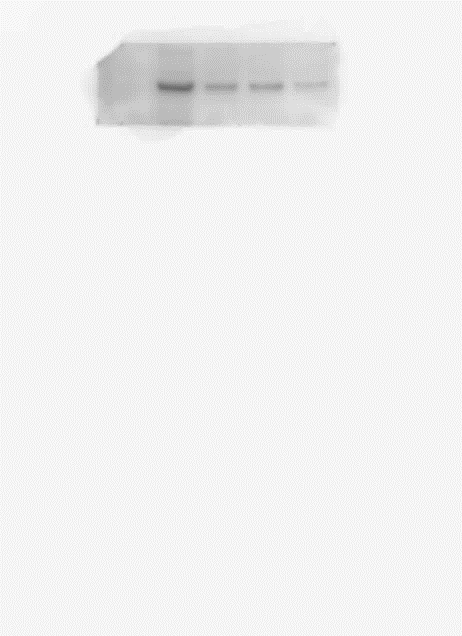

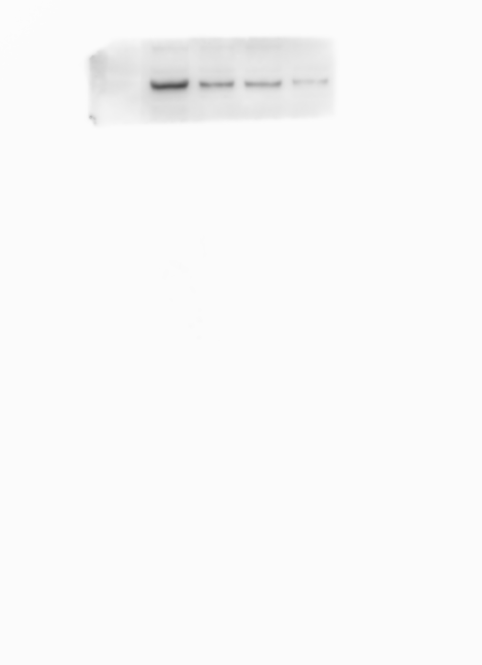


**Figure3-NANOG Figure3-Sox2**


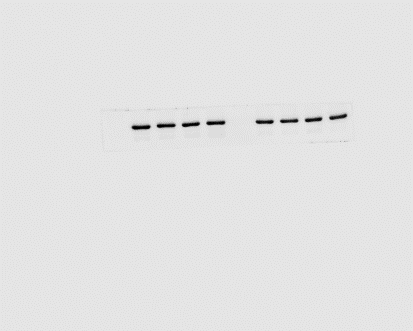


**Figure3 -GAPDH**

**Figure 4**

**G**


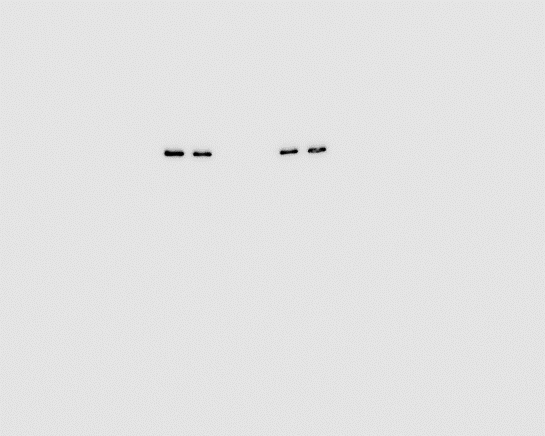


**Figure4-RIP**

**H**


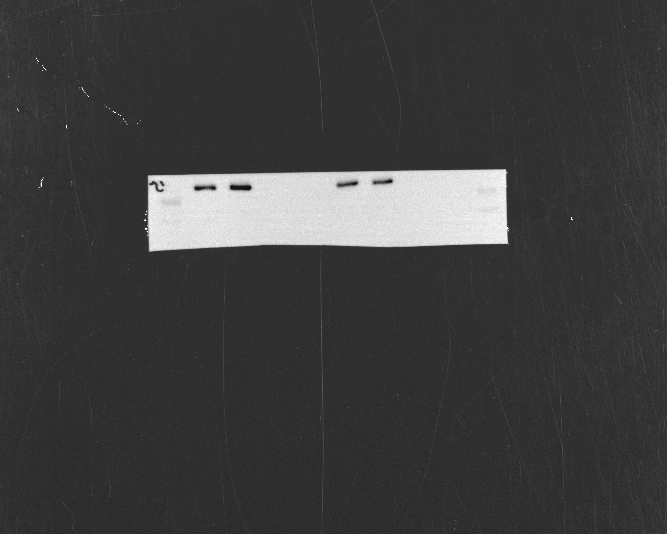


**Figure4-RNA PULLDOWN**

**Figure 5**

**F**


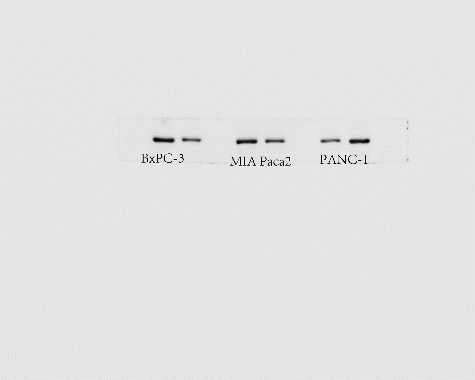

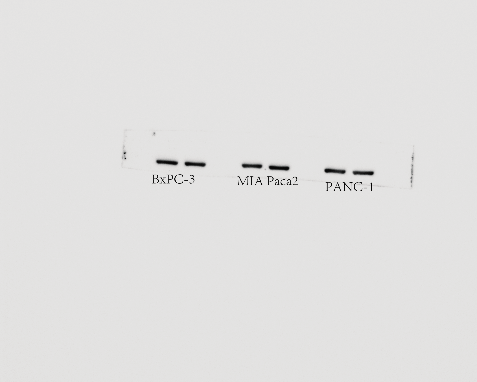


**Figure5-F-WNT5A Figure5-F-GAPDH**

**G**


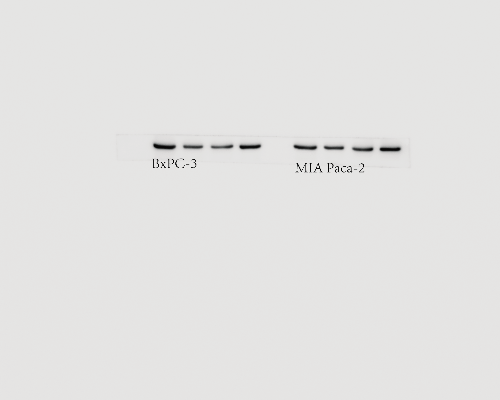

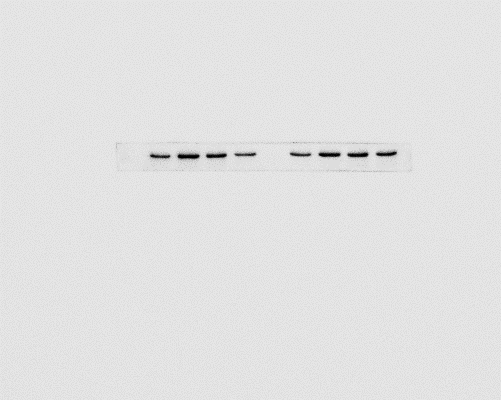


**Figure5-G-WNT5A Figure5-G-WNT5A-PANC-1**


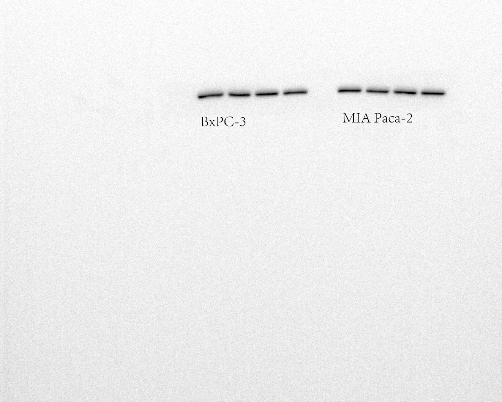

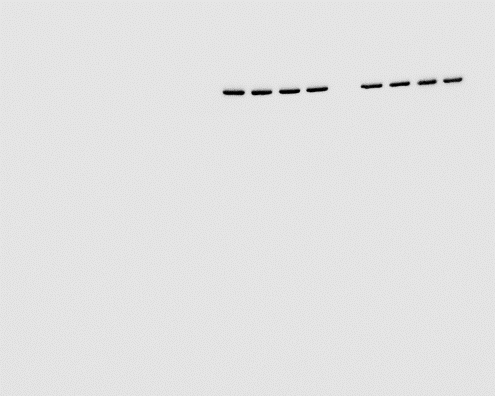


**Figure5-G-GAPDH Figure5-G-GAPDH-PANC-1**

**Figure 7**

**A**


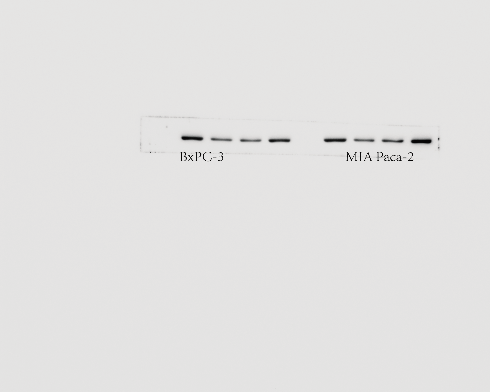

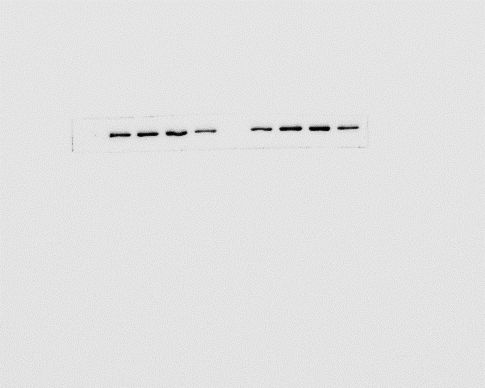


**Figure7-A-WNT5A Figure7-A-WNT5A-PANC-1**


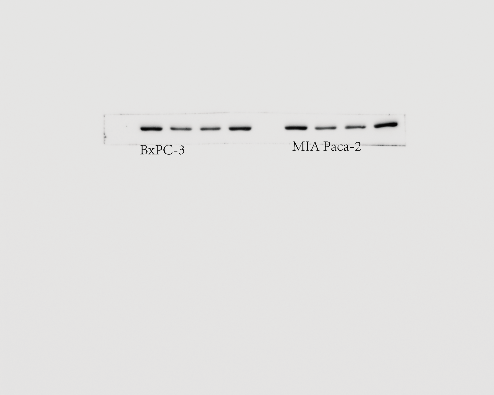

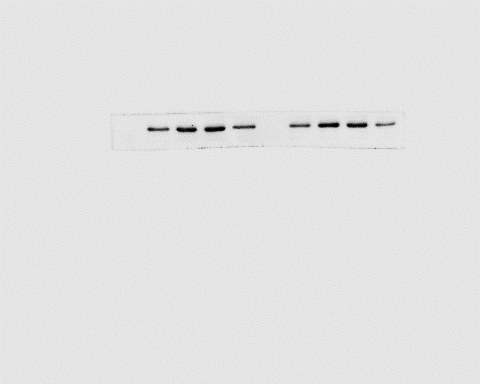


**Figure7-A-β-catenin Figure7-A-β-catenin-PANC-1**


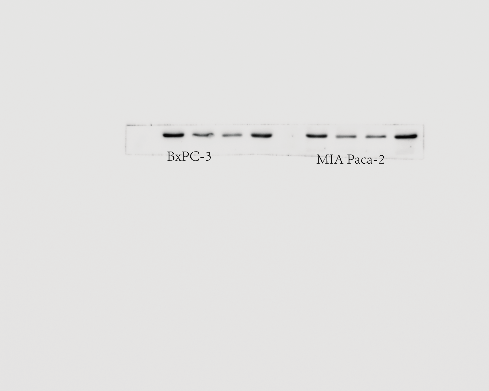

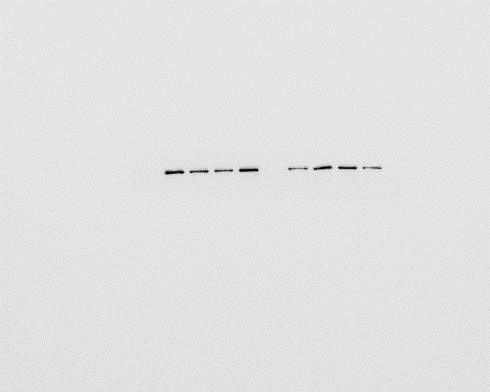


**Figure7-A-CyclinD1 Figure7-A-CyclinD1-PANC-1**


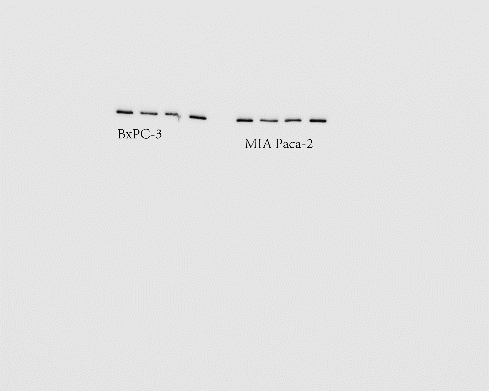

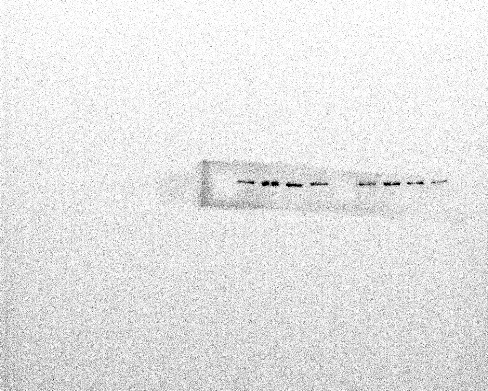


**Figure7-A-C-myc Figure7-A-C-myc-PANC-1**


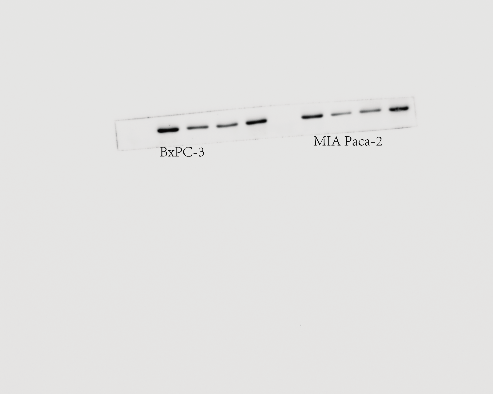

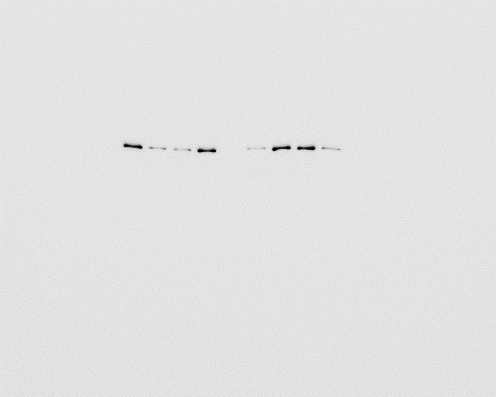


**Figure7-A-Sox2 Figure7-A-Sox2-PANC-1**


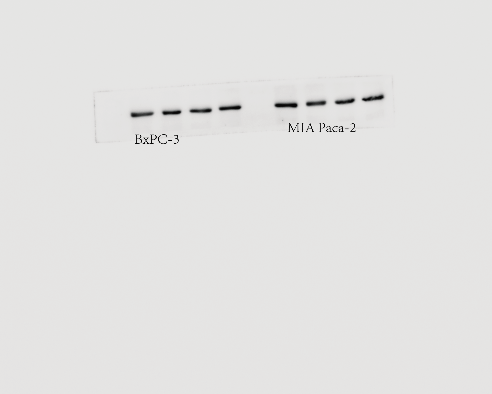

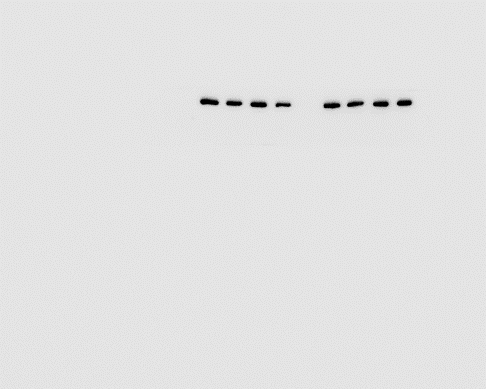


**Figure7-A-GAPDH Figure7-A-GAPDH-PANC-1**
